# Methionine Adenosyltransferase 1A and S-Adenosylmethionine in Alcohol-Associated Liver Disease

**DOI:** 10.3390/antiox14121486

**Published:** 2025-12-11

**Authors:** Lucía Barbier-Torres, Jyoti Chhimwal, José M. Mato, Shelly C. Lu

**Affiliations:** 1 Karsh Division of Gastroenterology and Hepatology, Cedars-Sinai Medical Center, Los Angeles, CA 90048, USA; lucia.barbier@gmail.com (L.B.-T.); jyoti.chhimwal@cshs.org (J.C.); 2 CIC bioGUNE, Centro de Investigación Biomédica en Red de Enfermedades Hepáticas y Digestivas (Ciberehd), Technology, Park of Bizkaia, 48160 Derio, Spain; director@cicbiogune.es

**Keywords:** alcohol-associated liver disease, methionine adenosyltransferase α1, S-adenosylmethionine, hypomethylation, oxidative stress, mitochondrial injury

## Abstract

Alcohol-associated liver disease (ALD) is a leading cause of liver-related morbidity, mortality, and premature death worldwide. Its pathogenesis is complex and incompletely understood, with disrupted methionine metabolism as a key contributor. This pathway converts methionine into S-adenosylmethionine (SAM or SAMe), the principal methyl donor, a precursor of glutathione (GSH), and a critical regulator of hepatocellular function. Alterations in methionine metabolism are primarily driven by downregulation of methionine adenosyltransferase 1A (*MAT1A*), the liver-specific gene encoding the MATα1 subunit responsible for SAMe biosynthesis. Reduced *MAT1A* expression and activity lead to hepatic SAMe and GSH deficiency, resulting in global hypomethylation, mitochondrial dysfunction, impaired lipid metabolism, and progressive liver injury, hallmarks of ALD. Recent studies show that MATα1 also localizes to hepatocyte mitochondria, where its selective depletion contributes to mitochondrial dysfunction in ALD. Experimental models demonstrate that SAMe supplementation restores methylation capacity, replenishes GSH, reduces oxidative stress, and improves mitochondrial function and liver histology. Preservation of mitochondrial MATα1 also protects against ALD, underscoring its importance in hepatocellular health. Clinical exploration of SAMe in early-stage ALD suggests potential benefit and motivates continued investigation into treatment strategies that build on and extend beyond supplementation. This review summarizes current knowledge on the role of the *MAT1A*/SAMe axis in ALD pathophysiology, emphasizing molecular functions and critically evaluating preclinical and clinical evidence for potential therapy.

## 1. Introduction

Alcohol-associated liver disease (ALD) is a major and rising global contributor to liver-related morbidity and mortality [1,2]. The pathogenesis of ALD is complex and multifactorial, and despite its high prevalence, effective therapies remain limited [3]. Methionine metabolism plays a central role in maintaining liver homeostasis, and disruption of this pathway is a key contributor to ALD progression.

### 1.1. Alcohol-Associated Liver Disease

ALD represents a growing public health concern, contributing substantially to the global disease burden and premature mortality [1,2]. While earlier studies suggested that moderate alcohol consumption might be beneficial, more recent evidence indicates that even low levels of intake can have serious health consequences [4,5]. Chronic alcohol consumption accounts for over 5% of the global disease burden (≈3 million cases), and about 50% of cirrhosis-related deaths are attributed to alcohol [6]. Accurate assessment of the impact of ALD is limited by underreporting and diagnostic challenges, but it is responsible for approximately 372,000 liver-related deaths worldwide each year [2]. Since the COVID-19 pandemic, ALD rates have increased across the globe, particularly among younger adults, and it is now the leading cause of liver transplantation in the United States and Europe, largely reflecting rising alcohol consumption [6].

ALD encompasses a broad clinical spectrum, ranging from simple hepatic steatosis to more severe and potentially life-threatening conditions. The earliest stage, alcohol-associated fatty liver, is characterized by hepatic fat accumulation and is often asymptomatic, although it can progress if alcohol consumption continues. Persistent drinking can lead to alcoholic steatohepatitis (ASH), marked by hepatocellular injury, inflammation, and varying degrees of fibrosis. With ongoing injury, fibrosis may advance to cirrhosis, which significantly increases the risk of liver failure and hepatocellular carcinoma (HCC) [3]. Among these stages, alcohol-associated hepatitis (AH) represents an acute and severe form, often presenting with high short-term mortality [7]. Disease progression is highly variable and influenced by the amount and duration of alcohol intake, genetic predisposition, sex, comorbidities such as obesity or viral hepatitis, and environmental factors [3,8].

Despite its high global burden, therapeutic options for ALD remain limited and current strategies primarily focus on alcohol abstinence—which can be difficult to achieve—nutritional support, and management of complications, with pharmacological interventions such as corticosteroids or liver transplantation reserved for severe cases [9]. Several therapeutic targets have been evaluated in clinical trials over the past decade; however, none have yet met the endpoints required for Food and Drug Administration (FDA) approval for routine clinical use. These strategies have included agents aimed at mitigating hepatocyte injury, such as antioxidants (e.g., N-acetylcysteine and metadoxine) [10,11] and liver-regenerative therapies (e.g., G-CSF and IL-22 analogs) [12,13,14], as well as inhibitors of hepatocyte death (e.g., selonsertib (NCT02854631)), although many have failed to show clinical benefit. Additional approaches have targeted hepatic inflammation using corticosteroids (NCT02281929) or anti-TNFα [15,16] or anti-IL-1 agents (NCT03775109 and NCT04072822), but results have been largely negative or of insufficient efficacy. More recently, therapies directed at the gut-liver axis—including FXR agonists (NCT02039219), probiotics (e.g., *Lactobacillus*) [17], fecal microbiota transplantation [18,19], and antibiotic regimens (NCT02281929)—have shown encouraging preliminary outcomes, yet remain under investigation. A comprehensive understanding of the clinical spectrum of ALD and its underlying mechanisms, along with improved public health initiatives, diagnostics, and research, is essential to reduce its growing prevalence and burden.

The pathogenesis of ALD is complex, multifactorial, and strongly influenced by disease stage. After intestinal absorption, ethanol is primarily metabolized in hepatocytes by alcohol dehydrogenase (ADH) to acetaldehyde—a highly reactive and toxic intermediate that forms adducts with proteins, nucleic acids, and lipids, impairing cellular functions and contributing to hepatotoxicity—and subsequently converted by aldehyde dehydrogenase (ALDH) to the less toxic acetate for elimination. Alcohol induces the expression and activity of certain cytochromes P450, particularly cytochrome P450 2E1 (CYP2E1), which also metabolizes ethanol while generating reactive oxygen species (ROS), exacerbating oxidative stress and hepatocellular injury; a minor pathway involving catalase also contributes to ethanol oxidation [20].

Ethanol oxidative metabolism causes a redox imbalance (nicotinamide adenine dinucleotide (NAD^+^/NADH ratio) and excess ROS production, leading to glutathione (GSH) depletion, lipid peroxidation, and mitochondrial damage [21]. These effects collectively trigger hepatocyte injury and activate inflammatory and fibrogenic responses that drive disease progression [20]. Concurrently, ethanol promotes hepatic steatosis by increasing de novo lipogenesis and fatty acid uptake, while inhibiting β-oxidation and lipid export via multiple mechanisms [22]. Mitochondrial dysfunction plays a central role in ALD by impairing energy production and fatty acid β-oxidation while also promoting the release of pro-inflammatory cytokines and activation of cell death pathways [23,24]. Alcohol activates multiple additional mechanisms that contribute to hepatocyte death, including the unfolded protein response, endoplasmic reticulum (ER) stress, danger-associated molecular patterns (DAMPs), and dysregulation of autophagy and membrane trafficking [8,25]. Moreover, various forms of hepatocyte death, including apoptosis, necroptosis, pyroptosis, and ferroptosis, coexist in ALD, underscoring the multifactorial nature of its pathogenesis [8].

Beyond hepatocytes, non-parenchymal liver cells, including Kupffer cells, liver-associated lymphocytes, liver sinusoidal endothelial cells, and hepatic stellate cells (HSCs), are also key contributors [26]. Chronic alcohol consumption triggers both innate and adaptive immune responses. Hepatocyte stress and death release damage-associated molecular patterns (DAMPs) that activate Kupffer cells, which produce pro-inflammatory cytokines (e.g., TNF-α, IL-1β) and chemokines, recruiting additional immune cells and amplifying inflammation [27]. Alcohol-induced dysbiosis and increased intestinal permeability allow bacterial products, such as lipopolysaccharide (LPS), to enter the portal circulation and further activate Kupffer cells [28,29]. This cascade recruits and stimulates additional immune cells—including neutrophils, natural killer (NK) and NKT cells, and T cells—exacerbating liver injury [29]. In addition to the gut-liver axis, the adipose tissue-liver axis is increasingly recognized in ALD, with adipose tissue releasing free fatty acids and adipokines that modulate hepatocyte metabolism and inflammation [30]. Persistent inflammation and oxidative stress activate HSCs, driving fibrosis [31,32]. These mechanisms establish a self-perpetuating cycle of hepatocyte injury, inflammation, and fibrogenesis that underlies ALD progression. Recent studies have also highlighted the role of non-coding RNAs, particularly microRNAs, in regulating liver injury and fibrosis in ALD [33]. Epigenetic modifications, including DNA methylation and histone modifications, further contribute to sustained inflammation and fibrosis [34].

Recent evidence highlights the circadian clock as a central regulator of hepatic physiology, integrating metabolic, inflammatory, and stress-response pathways [35,36]. The liver clock orchestrates daily rhythms in methionine and lipid metabolism, mitochondrial function, autophagy, cell-death pathways, and the fibrotic response—mechanisms involved in the development and progression of ALD. It also regulates xenobiotic detoxification and antioxidant defenses, thereby shaping the hepatic response to ethanol exposure [35,36]. Disruption of circadian rhythms has been shown to exacerbate steatosis, oxidative stress, inflammation, and fibrotic remodeling following alcohol intake [37,38]. Altogether, these findings position the circadian clock as an additional layer of regulation in ALD pathogenesis and underscore the importance of considering circadian control when evaluating therapeutic strategies that target metabolic, inflammatory, and stress-response pathways.

Although the full pathogenesis of ALD is not yet fully elucidated, oxidative stress and mitochondrial dysfunction—driven by metabolic alterations including impaired methionine metabolism—are central mechanisms that compromise hepatocellular viability, impair liver regeneration, and contribute to progressive liver damage.

This review is based on a targeted literature search conducted between 1980 and 2025 using PubMed. Keywords included “alcohol-associated liver disease,” “alcoholic liver disease,” “methionine metabolism,” “SAMe,” “MAT1A,” “oxidative stress,” “mitochondrial dysfunction,” “fibrosis,” “epigenetics,” and related terms. Studies were selected for inclusion based on their relevance to ALD pathogenesis, molecular and metabolic mechanisms, and emerging therapeutic insights. We prioritized peer-reviewed original research articles and high-quality reviews, while excluding non-English-language publications and non-peer-reviewed sources. As this is a narrative rather than a systematic review, no formal PRISMA methodology was applied; however, the search process is described here to enhance clarity and transparency.

### 1.2. Methionine Metabolism

Methionine, an essential sulfur-containing amino acid obtained from the diet, is primarily metabolized in the liver, where it serves as the precursor of S-adenosylmethionine (SAMe), the principal biological methyl donor [39]. SAMe is indispensable for cellular function, fueling a wide range of methylation reactions required for epigenetic regulation, RNA and protein methylation, phospholipid and neurotransmitter synthesis, and the generation of key metabolites such as creatine, carnitine, and GSH. In addition, methionine itself serves as the initiating amino acid for protein synthesis, making its metabolism critical for fundamental processes, from gene expression to cellular growth and stress responses [40].

The first step of methionine metabolism involves its conversion, together with adenosine triphosphate (ATP), into SAMe by methionine adenosyltransferases (MATs). There are three MAT genes in mammals: *MAT1A* and *MAT2A* encode for catalytic subunits of MAT isoenzymes, and *MAT2B* encodes for a regulatory subunit, and their expression in the liver follows a developmental and cell-type-specific pattern [39]. MAT2A predominates in fetal liver, whereas MAT1A becomes the main isoform in adult liver (mostly hepatocytes). MAT2A and MAT2B are expressed in extrahepatic tissues and non-parenchymal hepatic cells (HSCs and Kupffer cells). In proliferating or de-differentiated hepatocytes and in settings of liver injury, fibrosis, and cancer, a switch occurs, with MAT1A downregulation and MAT2A/MAT2B re-expression [41].

*MAT1A* encodes MATα1 (396 amino acids), which exists as a tetramer (MATI) or a dimer (MATIII), while *MAT2A* encodes MATα2 (395 amino acids), which forms MATII, a less efficient isoenzyme for regulating SAMe levels. *MAT2B* encodes MATβ (334 amino acids) interacts with MATα2, lowering the inhibition constant (K_i_) for SAMe and the Michaelis constant (K_m_) for methionine, thereby fine-tuning MATII activity and SAMe production in response to metabolic demands [39]. For simplicity, MAT1A and MAT2A will refer to both the genes and the isoenzymes they encode hereinafter.

**Figure 1:**
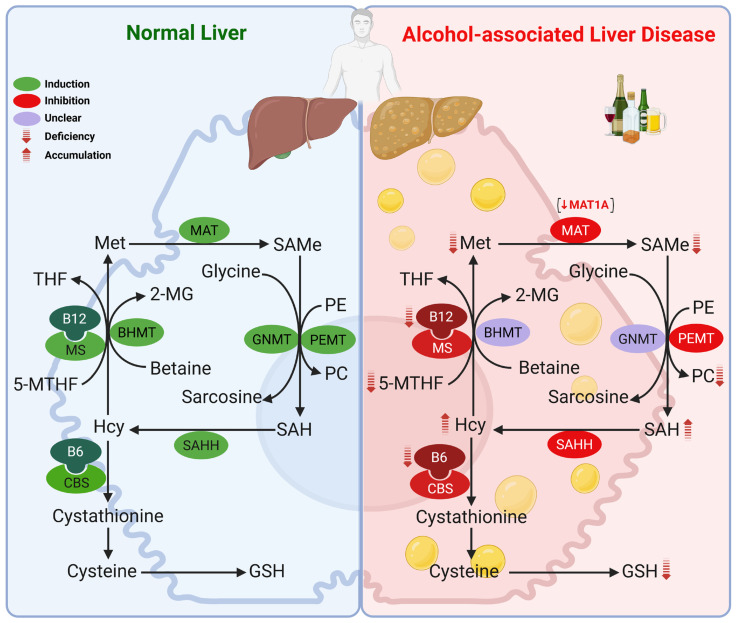
Methionine metabolism in the healthy liver and its alterations in ALD. Chronic alcohol consumption disrupts multiple enzymatic steps, leading to decreased methionine, SAMe, and GSH hepatic levels, with concomitant accumulation of SAH and homocysteine, resulting in a reduced SAMe/SAH ratio. These changes are primarily driven by downregulation of MAT1A, along with decreased expression or activity of MS, CBS, and SAHH. Reduced folate and vitamin B_6_/B_12_ further exacerbate these abnormalities. A reduced SAMe/SAH ratio also impairs PC synthesis via the PEMT pathway. Abbreviations: 2-MG: 2-methylglycine; 5-MTHF: 5-methyltetrahydrofolate; B12: vitamin B12; B6: vitamin B6; BHMT: betaine-homocysteine methyltransferase; CBS: cystathionine β-synthase; GMNT: glycine N-methyltransferase; GSH: glutathione; Hcy: homocysteine; MAT: methionine adenosyltransferase; Met: methionine; MS: methionine synthase; PC: phosphatidylcholine; PE: phosphatidylethanolamine; PEMT: phosphatidylethanolamine methyltransferase; SAH: S-adenosylhomocysteine; SAHH: SAH hydrolase; SAMe: S-adenosyl-methionine; THF: tetrahydrofolate.

After donating its methyl group, SAMe is converted to S-adenosylhomocysteine (SAH), a potent inhibitor of methylation reactions. The SAMe/SAH ratio is a well-recognized metabolic indicator of cellular methylation capacity, with a reduced ratio reflecting impaired methylation potential [42]. In hepatocytes, glycine N-methyltransferase (GNMT)—the predominant hepatic methyltransferase—plays a central role in regulating this step [43,44,45]. By catalyzing the methylation of glycine to sarcosine, GNMT acts as a methylation buffer, preventing excessive SAMe accumulation and helping to preserve hepatic methylation potential within a physiological range [46,47,48,49,50]. Importantly, GNMT is inhibited by 5-methyltetrahydrofolate (5-MTHF), the active form of folate, while sarcosine demethylation produces methylene-THF, which can be converted to 5-MTHF. The removal of SAH is equally critical, as its intracellular accumulation would inhibit methylation reactions. This reaction is catalyzed by S-adenosylhomocysteine hydrolase (SAHH), which hydrolyzes SAH to homocysteine and adenosine, thereby sustaining the SAMe/SAH ratio and permitting ongoing transmethylation reactions. Homocysteine can then follow one of three pathways: it may be remethylated back to methionine via methionine synthase (MS), which requires 5-MTHF and vitamin B_12_ as cofactors, or via betaine-homocysteine methyltransferase (BHMT), which uses betaine as the methyl donor; alternatively homocysteine can form homocysitaconate through conjugation with itaconate, a reaction catalyzed by S-adenosyl-L-homocysteine hydrolase [51] SAMe binds to a distinct “reactivation domain” of MS, a site required to restore the enzyme’s active form through a cycle involving methionine synthase reductase (MSR). In this reaction, SAMe donates a methyl group to the inactive enzyme, regenerating its active, methylated state. Alternatively, homocysteine may enter the transsulfuration pathway, where cystathionine β-synthase (CBS)—an enzyme activated by SAMe—converts it to cystathionine in a vitamin B_6_-dependent reaction, ultimately yielding cysteine for GSH synthesis [52,53]. This tightly regulated cycle integrates methylation capacity, redox balance, and sulfur amino acid metabolism, making it essential for cellular function and liver homeostasis (Figure 1).

Alterations in methionine metabolism play a central role in the pathogenesis of ALD, with hepatic SAMe deficiency first described in the early 1980s [54]. Our group and others have shown that alcohol disrupts methionine metabolism at multiple enzymatic steps, leading to decreased hepatic methionine, SAMe and GSH, accumulation of SAH and homocysteine (hyperhomocysteinemia), and a decline in the SAMe/SAH ratio [55,56,57,58,59,60,61]. This has been observed in livers from rats fed ethanol intragastrically [55,61], or using the Lieber-DeCarli diet [57,59], in baboons chronically fed ethanol [58], and in AH patients [60]. The importance of SAMe deficiency in ALD is underscored by numerous studies showing that SAMe supplementation ameliorates alcohol-induced liver injury in experimental models, including rats and baboons [58,62,63,64,65,66,67,68] as well as in humans [69]. This deficiency is largely attributed to MAT1A downregulation, involving alterations in its mRNA levels, enzymatic activity, and protein stability. A detailed overview of MAT1A is provided in Section 2.2. A switch toward MAT2A was observed in ethanol-fed rats and likely contributes to impaired hepatic SAMe levels [55].

Beyond MAT1A, other enzymes in the pathway are altered in ALD, with several studies reporting reduced expression and activity of MS and CBS. As shown in Figure 1, impaired MS and MAT1A activity reduces SAMe synthesis, while loss of MS and CBS activity contributes to elevated homocysteine. Increases in SAH and a reduced SAMe/SAH ratio result from either diminished forward flux through SAHH or excess homocysteine driving the reverse reaction. Consistent with these mechanisms, reduced hepatic expression of MS, MAT1A, and CBS in patients with AH [60] and alcohol-associated cirrhosis [70] have been documented. Ethanol-fed micropigs likewise exhibited decreased expression and activity of MS, MAT1A, and SAHH [71] in the liver, while in rats, chronic ethanol feeding reduced MS activity with compensatory induction of BHMT [56,57,72,73]. In contrast, mRNA levels of BHMT were markedly lower in the livers of patients with alcoholic cirrhosis [70]; highlighting the complexity of methionine resynthesis and homocysteine catabolism across models and disease stages. *GNMT* mRNA levels were reduced in the livers of AH [60] and alcohol-associated end-stage cirrhotic patients [74], but increased in the livers of ethanol-fed micropigs [71], although its role in ALD remains unclear. Deficiencies in folate, vitamin B_6_, and vitamin B_12_ are also common in ALD [75] and may lead to alterations in this pathway as well, since they are essential cofactors for MS (folate and B_12_) and CBS (B_6_). Although glutamate-cysteine ligase catalytic subunit (GCLC) is induced in livers of rats fed ethanol [76], its expression, along with that of glutathione synthetase (GS), the second enzyme in GSH synthesis, is decreased by 50% in livers of AH patients [60], suggesting that the decrease in hepatic GSH levels in ALD is likely multifactorial, including nutritional deficiency, decreased precursor availability (SAMe), and decreased capacity to synthesize GSH.

Collectively, these findings demonstrate the central role that alterations in the methionine metabolism pathway play in the onset and progression of ALD. In this review, we will focus on the effects of reduced MAT1A and SAMe levels in ALD, which will be discussed in more detail in the following sections.

## 2. The MAT1A–SAMe Axis in Liver Physiology

### 2.1. S-Adenosylmethionine

SAMe is a molecule present in all cells, tissues and body fluids that is indispensable for life as the primary donor of methyl groups. It drives a wide range of transmethylation reactions that are essential for DNA, RNA, protein, lipid, and small molecule methylation [39,77]. In addition, SAMe contributes to polyamine synthesis, supporting cell growth and differentiation, to antioxidant defense as a precursor for GSH, and is also required for the biosynthesis of key metabolites such as creatine, carnitine, and phosphatidylcholine (PC) [39]. Through these pathways, SAMe regulates critical processes including epigenetic regulation, gene expression, energy and lipid metabolism, membrane fluidity, and signal transduction. Because of its central role in sustaining methylation, redox balance, and biosynthetic functions, SAMe is vital for maintaining cellular homeostasis and is especially crucial for liver physiology [39,77].

Although SAMe is present in all cells, its levels in the liver must be tightly regulated, as roughly half of the daily methionine intake is converted into SAMe in the liver, and up to 85% of transmethylation reactions take place there, making it a key regulator of hepatic metabolism [78,79]. Until recently, SAMe was thought to be synthesized exclusively in the cytosol and then transported to other cellular compartments. However, the discovery of MATα1 in the nucleus [80] and mitochondria [81] of hepatocytes suggests that SAMe can also be produced locally in these organelles to meet distinct metabolic and regulatory needs.

In healthy adult liver, SAMe concentrations are tightly regulated by MAT1A and GNMT, ensuring an adequate supply to meet diverse metabolic demands without excessive accumulation. Proper SAMe levels are essential for preserving mitochondrial function, supporting hepatocyte survival and proliferation, and ultimately, sustaining normal liver structure and function. On this note, while SAMe plays an anti-apoptotic role in hepatocytes, partly by raising the antioxidant capacity as a GSH precursor and suppressing the induction of TNF-α [82,83], it induces apoptosis in cancer cells by different mechanisms like selectively inducing Bcl-x_S_–the pro-apoptotic variant of Bcl-x [84], further protecting liver health.

Disruption of SAMe homeostasis can result in either abnormally low or excessively high SAMe levels, both of which are detrimental to liver physiology. This has been observed in genetic mouse models targeting key regulators of methionine metabolism. Mice lacking *Mat1a* (*Mat1a*-KO) exhibit chronically reduced hepatic SAMe levels, are more susceptible to steatosis and liver injury, and spontaneously develop steatohepatitis and HCC [85,86]. These mice have increased hepatic oxidative stress—partly due to reduced GSH [85] levels and upregulation of CYP2E1 [81,86]—along with abnormal lipid homeostasis, impaired mitochondrial function—partly due to reduced prohibitin 1 (PHB1) expression [87]— increased protein phosphorylation [88] and SUMOylation [89], increased genomic instability [90], expansion of liver stem cells as they age [91], and dysregulated ERK [92] and LKB1/AMPK [93] signaling, among others. The *Mat1a*-KO mouse model is highly relevant to human liver disease, as MAT1A expression is markedly reduced in most patients with ALD, metabolic dysfunction-associated steatotic liver disease (MASLD), cirrhosis and HCC, and recapitulates the metabolic profile observed in a large subset (~40–50%) of MASLD patients [94]. A study from Alarcon-Vila C. et al. showed that these mice are also more susceptible to ALD [95].

Conversely, mice deficient in *Gnmt* (*Gnmt*-KO) are unable to regulate SAMe disposal through glycine methylation, leading to persistently elevated hepatic SAMe levels. This results in global DNA hypermethylation, disrupted gene expression, and metabolic imbalance, which together promote liver injury, fatty liver, and a strong predisposition to HCC development [50]. Patients with GNMT mutations have also been reported to present with chronic liver injury [96]. Taken together, hepatic SAMe levels must be tightly regulated to preserve normal liver function and overall health.

Reduced hepatic SAMe levels are a consistent finding in both experimental models and patients with ALD and have direct pathogenic consequences that contribute to disease progression [55,57,58,60,62,66,67,97,98,99,100,101,102] (Table 1). Low SAMe decreases the SAMe/SAH ratio, thereby inhibiting transmethylation reactions and causing global DNA and gene-specific hypomethylation that alter hepatocyte gene expression. SAMe deficiency also impairs transsulfuration and the synthesis of GSH, limiting antioxidant defenses and increasing susceptibility to oxidative stress. Importantly, SAMe directly activates CBS, the first enzyme of the transsulfuration pathway, linking methylation capacity to GSH production beyond simply providing sulfur atoms [103]. At the mitochondrial level, reduced SAMe availability compromises protein methylation [104,105] and disrupts electron transport chain (ETC) activity [62,67,105], and increases CYP2E1-mediated ROS production [81], altogether undermining mitochondrial respiration and integrity. Furthermore, a reduced SAMe/SAH ratio impairs PC synthesis via the phosphatidylethanolamine methyltransferase (PEMT) pathway, exacerbating hepatic steatosis [59,106]. Lastly, SAMe deficiency also disrupts proteostasis, as methylation-dependent proteasomal degradation of proteins such as CYP2E1 is diminished [81].

In summary, due to its central role in hepatic metabolism, ethanol-induced SAMe depletion disrupts multiple processes in the liver—including methylation, redox balance, lipid metabolism, and protein homeostasis—leading to oxidative stress, mitochondrial dysfunction, lipid accumulation, apoptosis, and inflammation, which collectively contribute to ALD pathogenesis. The most relevant molecular mechanisms by which SAMe deficiency to ALD are described in Section 3.

### 2.2. Methionine Adenosyltransferase 1A

Because MAT1A is the MAT gene expressed predominantly in adult hepatocytes, alterations in its expression or encoded enzyme activity can profoundly disrupt hepatic metabolism, function, and health [39]. As mentioned in the previous section, the intracellular localization of MATα1 is not limited to the cytosol; it is also present in the nucleus [80] and mitochondria [81] of hepatocytes, suggesting compartment-specific roles and localized SAMe synthesis. Proper subcellular localization is therefore critical.

In the nucleus, active MATα1 supports local SAMe synthesis and correlates with increased histone H3K27 trimethylation, an epigenetic modification associated with gene repression [80]. Monomeric MATα1 is also found in nuclei and may participate in additional interactions [80,107]. Beyond its canonical enzymatic role, MATα1 functions as a transcriptional cofactor in hepatocytes, directly interacting with transcription factors such as c-MYC, MAX, c-MAF, and MAFG, along with PHB1, to modulate gene expression [108].

In mitochondria, MATα1 interacts with multiple proteins involved in key mitochondrial metabolic pathways, including the tricarboxylic (TCA) cycle, oxidative phosphorylation (OXPHOS), and fatty acid β-oxidation [81,104]. Its presence in mitochondria is linked to enhanced protein methylation. Our group found that in hepatocyte mitochondria, MATα1 interacts with and methylates CYP2E1, promoting its proteasomal degradation and thereby protecting against oxidative stress [81]. Although this field of research is relatively recent and incompletely understood, these findings indicate that mitochondrial MATα1, by providing localized SAMe, plays a crucial role in sustaining mitochondrial respiration and integrity, supporting protein methylation and redox homeostasis, and likely contributing to mtDNA stability and transcription.

Regulation of MAT1A expression occurs at transcriptional, post-transcriptional, and post-translational levels. The *MAT1A* promoter contains binding sites for multiple transcription factors, including hepatocyte nuclear factor (HNF), CCAAT enhancer binding protein (C/EBP), activator protein 1 (AP-1), and glucocorticoids; with HNF and C/EBP driving its liver-specific expression [109,110]. In a normal liver, *MAT1A* is upregulated via promoter hyperacetylation and cytosine hypomethylation [111]. PHB1, which is highly expressed in normal hepatocytes, positively regulates *MAT1A* mRNA levels, and MATα1 reciprocally regulates PHB1 [87,108]. Conversely, several oncogenes such as c-MYC, MAX, MAFG and c-MAF bind to E-box elements in the *MAT1A* promoter to repress its transcription [108,112,113]. AU-rich RNA binding factor (AUF1) binds the 3′-untranslated region to negatively regulate *MAT1A* mRNA stability [114]. In HCC, MAT1A is often downregulated by promoter hypermethylation and microRNAs, including miR-22, mir-29b, miR-485-3p, miR-495, and miR-664 [115,116]. Because *MAT1A* transcription is regulated by methylation, endogenous SAMe level can indirectly modulate its expression. However, short-term exogenous SAMe treatment raised MAT1A level in vitro (in MzChA-1 and HepG2 cells) and in vivo (mouse livers) [112,117]. The underlying mechanisms are likely indirect, as SAMe treatment can enhance binding of PHB1 while lowering binding of repressors such as c-MYC and MAFG to the *MAT1A* promoter [112].

At the protein level, post-translational modifications, including phosphorylation, SUMOylation, oxidation, and nitrosylation influence *MAT1A*-encoded enzyme activity, stability, subcellular localization, and oligomerization. Oxidation or nitrosylation of cysteine 121 (C121 in mice, C120 in humans), located in the flexible gating loop over the active site, disrupts its dimer–tetramer equilibrium and inactivates the enzyme [118,119,120]. This sensitivity to oxidative modifications links MATI/III activity to cellular redox status: adequate GSH levels support MATI/III function, whereas oxidative stress, ROS, and nitric oxide (NO) can inhibit enzymatic activity. Consequently, low SAMe leads to reduced GSH synthesis, generating oxidative stress that further inhibits MATI/III, forming a vicious cycle that amplifies SAMe deficiency and disrupts both methylation and antioxidant defenses.

Phosphorylation at threonine 342 (T342 in mice, T341 in humans) by protein kinase C (PKC) does not affect enzymatic activity, but dephosphorylation by alkaline phosphatase lowers the activity of both MATI and MATIII [121]. Other phosphorylation sites include serine 114 (S114 in humans, S115 in mice) by casein kinase 2 (CK2) [104], which enhances interaction with peptidyl-prolyl isomerase NIMA 1 (PIN1) and blocks MATα1 mitochondrial targeting; and serine 180 (S180 in humans, S181 in mice) and threonine 202 (T202 in humans, T203 in mice) by AKT, which promote interaction with 14-3-3ζ and prevent MATα1 nuclear targeting in HCC [122]. Large-scale phosphoproteomic studies have identified additional sites, but their functional consequences are uncharacterized.

Regarding SUMOylation, Floris et al., reported that MATα1 is SUMOylated by SUMO2 at lysine 48 (K48, K47 in humans), enhancing interaction with PIN1 and inhibits mitochondrial localization [89].

Lastly, the MATα1 interactome is extensive, and interactions with certain partners can alter its expression, activity, and localization. Some binding partners include PIN1 [104] and 14-3-3ζ [122], described above, as well as p53 and DNA damage-regulated gene 1 (PDRG1) [107]; the latter interacts with MATα1 in the nucleus, resulting in decreased MAT activity and DNA hypomethylation in hepatoma cells.

Some of these modifications are observed in ALD. Ethanol decreases mRNA via promoter hypermethylation, as observed in liver biopsies from patients with AH [60] and alcohol-associated cirrhosis [70]. Accordingly, MATα1 protein levels are also reduced in the livers of ALD patients. Oxidative stress and endotoxemia in ALD are likely to inactivate MATα1 through nitrosylation of cysteine 121. Although *MAT1A* mRNA changes are not seen in ethanol-fed mice, NIAAA diet-fed mice exhibit reduced MATα1 protein levels [81,89,104] and alcohol-fed baboons show loss of MAT1A activity [123]. More recently, we found that ethanol promotes MATα1 S114 phosphorylation and K47 SUMOylation, leading to mitochondrial depletion and proteasomal degradation of MATα1 in mice fed the NIAAA diet [89,104]. Mechanisms regulating MAT1A in healthy liver and ALD are summarized in Table 2.

## 3. Molecular Mechanisms Linking MAT1A and SAMe to ALD Pathogenesis

**Figure 2:**
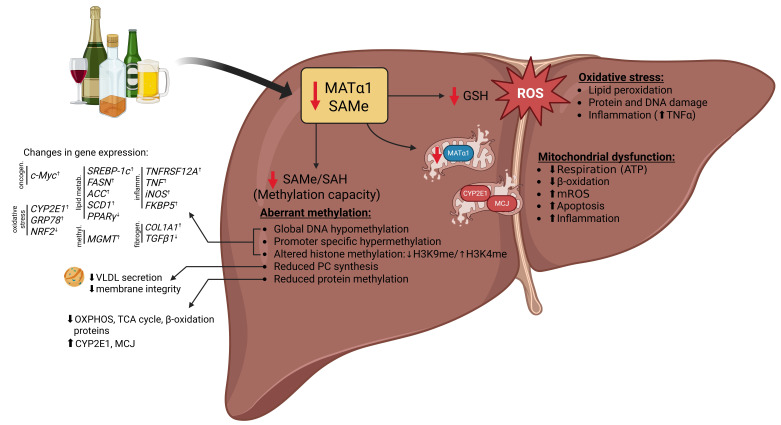
Consequences of MAT1A and SAMe downregulation in ALD pathogenesis. Loss of MAT1A and consequent depletion of hepatic SAMe have wide-ranging effects that contribute to the development and progression of ALD. Reduced SAMe levels impair transmethylation reactions, leading to global DNA and protein hypomethylation, as well as alterations in histone methylation and hypermethylation of specific gene promoters, resulting in aberrant gene expression, and disrupted phospholipid homeostasis. SAMe deficiency also limits GSH synthesis, enhancing oxidative stress and lipid peroxidation and rendering hepatocytes more susceptible to alcohol-induced injury. In parallel, mitochondrial dysfunction further promotes hepatocellular damage, inflammation, and fibrogenesis. Collectively, these events establish SAMe deficiency as a central driver of metabolic, epigenetic, and redox imbalances in ALD. Abbreviations: ATP: adenosine triphosphate; GSH: glutathione; MATα1: methionine adenosyltransferase α1; MCJ: methylation-controlled J-protein; mROS: mitochondrial reactive oxygen species; OXPHOS: oxidative phosphorylation; PC: phosphatidylcholine; ROS: reactive oxygen species; SAH: S-adenosylhomocysteine; SAMe: S-adenosyl-methionine; TCA: tricarboxylic acid; TNFα: tumor necrosis alpha; VLDL: very low-density lipoprotein. ↑ represents increase and ↓ represents decrease in levels.

Having outlined the central roles of MAT1A and SAMe in liver health, it is important to understand how their dysregulation contributes specifically to ALD. The following section summarizes the molecular pathways through which MAT1A loss and SAMe depletion drive ALD progression (Figure 2).

### 3.1. Aberrant Methylation

A hallmark consequence of disrupted methionine metabolism in ALD is reduced global methylation capacity, which impairs multiple hepatic methylation reactions and alters gene expression, protein function, and cellular homeostasis. One major outcome of a decreased SAMe/SAH ratio is global DNA hypomethylation, which affects genes relevant to ALD pathogenesis [75,77,124] (Figure 2). DNA hypomethylation has been reported in livers from various in vivo models, including ethanol-fed rats (accompanied by increased DNA strand breaks) [55], micropigs [125], alcohol-associated cirrhotic patients [70], and HCC patients with a history of alcohol consumption [126]. In livers from ethanol-fed mice, hypomethylation was observed across all 19 autosomes, primarily within gene bodies [127]. Interestingly, livers from patients with chronic alcohol abuse showed enhanced hypomethylation in pathways linked to alcohol dependence and alcohol abuse, patterns not observed in HCC of hepatitis C etiology [128].

Although few studies have examined specific genes, several examples highlight the functional impact of hypomethylation in ALD. In livers from ethanol-fed rats, *Nos2* (gene encoding the inducible nitric oxide synthase (iNOS) enzyme, which is responsible for producing NO) is hypomethylated at CpG sites [127], correlating with increased expression that promotes steatosis and necroinflammation through NF-κB activation and TNFα induction [129]. *c-Myc* hypomethylation leads to increased expression and potential predisposition to malignant transformation [55]. In humans, *ALDH2* hypomethylation has been reported in livers from alcohol-associated cirrhosis [130], while tumor necrosis factor receptor superfamily member 12A (*TNFRSF12A*) hypomethylation in alcohol-related HCC correlates with increased expression and poor prognosis [131]. Similarly, aberrant methylated-DNA–protein-cysteine methyltransferase (*MGMT*) hypomethylation has been linked to alcohol intake in HCC tumors [126]. In livers from ethanol-fed micropigs, transcripts of *CYP2E1*, ER stress genes (e.g., *GRP78*), the lipogenic transcription factor sterol regulatory element-binding protein 1 (*SREBP-1c*), and lipogenic enzymes (fatty acid synthase (*FASN*), acetyl-CoA carboxylase (*ACC*), stearoyl-CoA 9-desaturase (*SCD1*)) negatively correlate with the SAMe/SAH ratio [132,133]. Additionally, FK506-binding protein 51 (*FKBP5*) expression is upregulated through hypomethylation at its 5′ untranslated region (UTR) promoter region, likely affecting multiple stress-related pathways, as observed in livers from ethanol-fed mice [134].

Although global hypomethylation is a hallmark of ALD, promoter-specific hypermethylation has also been reported in ALD cirrhotic patients [135]. Increased global 5-methylcytosine (5mC) levels and upregulation of DNA methyltransferase 1 (*DNMT1*) and *DNMT3B* have been reported in liver biopsies from patients with AH [136], together with promoter hypermethylation of genes implicated in oxidative stress responses (*NRF2*), lipid metabolism (*PPARγ*), and fibrogenesis (*TGFβ1*, *COL1A1*). Although more studies are needed to clarify how hypermethylation at certain genes occurs in the setting of global hypomethylation and how these aberrant methylation patterns contribute to disease progression, current evidence indicates that restoring the SAMe/SAH ratio normalizes DNA methylation and reverses ethanol-induced gene expression changes, thereby attenuating liver injury [56,127,137]. It is plausible that the coexistence of global DNA hypomethylation and promoter-specific hypermethylation in ALD reflects a selective redistribution of methylation activity rather than a uniform loss across the genome. Alcohol-induced disruption of one-carbon metabolism and the consequent decrease in the SAMe/SAH ratio may globally limit methylation capacity; however, DNMTs may remain active and be selectively recruited to specific promoters. This targeted hypermethylation could be facilitated by alterations in chromatin structure, histone modifications, or transcription factor occupancy induced by oxidative stress and inflammation. Thus, in the setting of reduced methyl donor availability, the methylation machinery may be differentially directed toward certain regulatory regions, leading to localized hypermethylation despite an overall hypomethylated genomic landscape.

Histone methylation is also disrupted by SAMe depletion [138]. Reduced SAMe shifts the balance between activating (H3K4me2/3) and repressive (H3K9me2/3, H3K27me3) histone marks, altering chromatin structure and transcriptional regulation in hepatocytes [139,140]. Ethanol decreases H3K9 methylation while increasing H3K4 methylation, correlating with upregulation of ethanol-responsive genes such as ADH1 and glutathione S-transferase (GST)-Yc2 and downregulation of L-serine dehydratase and CYP2C11 [139]. Chronic ethanol feeding also increases H3K27me3 in hepatocytes, selectively repressing certain gene subsets [141]. SAMe supplementation restores histone methylation, stabilizes gene expression, and attenuates liver injury. An example of this is decrease TNFα expression in LPS-stimulated Kupffer cells at the level of histone methylation after SAMe treatment [83,142].

RNA methylation, particularly m6A, may also be altered in ALD, potentially influencing mRNA stability, splicing, and translation, although direct evidence is limited. Ethanol-induced promoter hypomethylation upregulates miR-34a in ethanol-fed mice and ethanol-treated mouse hepatocytes, which in turn targets caspase 2 (CASP2), sirtuin 1 (SIRT1), matrix metallopeptidase 1 (MMP1), and MMP2, thereby promoting apoptosis and hepatic remodeling [143]. Collectively, alcohol-induced alterations in DNA, histone, and RNA methylation disrupt epigenetic programming and contribute to ALD pathogenesis.

In HSCs, disrupted methylation directly contributes to their activation and fibrogenic response. DNA hypomethylation and altered histone marks, such as decreased H3K9me3 and increased H3K4me3 at fibrogenic gene promoters, enhance the expression of transforming growth factor-beta 1 (TGF-β1), platelet-derived growth factor (PDGF), and collagen genes [144]. In vitro studies using HSCs [116,119] and LX-2 [117] cells, as well as in vivo mouse experiments [119], show that SAMe suppresses HSC activation and profibrotic gene expression [145,146,147,148], linking impaired methylation to fibrosis in ALD.

Aberrant methylation also extends to lipids, particularly PC, which requires methylation of phosphatidylethanolamine (PE) via the PEMT pathway [106]. SAMe deficiency in ALD reduces PC synthesis, disrupting membrane integrity, very-low-density lipoprotein (VLDL) secretion, and lipid export. Impaired lipid methylation exacerbates hepatic steatosis by promoting intracellular lipid accumulation and sensitizing hepatocytes to lipotoxic stress [59,149].

Lastly, protein methylation is similarly compromised in ALD. Reduced hepatic SAMe limits protein methylation, affecting function and stability. Alcohol-induced depletion of mitochondrial MATα1 has been observed in ethanol-fed mice and ethanol-treated hepatocytes, which decreases SAMe availability within mitochondria, leading to hypomethylation of proteins involved in the TCA cycle, OXPHOS, and fatty acid β-oxidation [104]. Furthermore, reduced methylation of CYP2E1 at residue R379 has been observed in mouse livers and correlates with increased mitochondrial CYP2E1 levels, enhancing oxidative stress and mitochondrial injury [81]. Similarly, we observed that SAMe regulates the methylation of the methylation-controlled J (MCJ), an endogenous inhibitor of the ETC that is upregulated in ALD, leading to reduced expression in hepatocytes [150]. Preserving mitochondrial MATα1 restores protein methylation and improves hepatocyte function, highlighting the importance of SAMe-dependent protein methylation in maintaining mitochondrial integrity and its perturbation in ALD [81,104]. In line with these findings, Schober et al. [105] demonstrated in Drosophila models and *Slc25a26*-deficient mouse embryonic fibroblasts that mitochondrial methylation potential is essential for energy metabolism, as reduced mitochondrial SAMe impairs methylation of complex I proteins and Fe-S clusters, decreasing their stability and functionality, and compromising OXPHOS and ATP production [105]. Methylation of mitochondrial rRNA and tRNA is essential for protein synthesis and mitochondrial function [151], and disruption of these modifications can impair translation, reduce activity of the ETC, and lead to energy deficits and oxidative stress. Although not directly studied in ALD, these events likely contribute to mitochondrial dysfunction in ethanol-exposed hepatocytes.

Overall, these widespread alterations in DNA, histone, RNA, lipid, and protein methylation underscore the central role of SAMe-dependent methylation in maintaining hepatic homeostasis. Their dysregulation underlies steatosis, oxidative stress, inflammation, fibrosis, and mitochondrial dysfunction in ALD, emphasizing methylation defects as key drivers of disease progression.

### 3.2. Oxidative Stress

Oxidative stress is a major driver of ALD, resulting from the imbalance between excessive generation of ROS and reactive nitrogen species (RNS), and impaired antioxidant defenses [21]. Ethanol metabolism via ADH, CYP2E1, and catalase produces acetaldehyde and ROS, including superoxide anions and hydrogen peroxide, which overwhelm hepatocellular redox homeostasis [20]. CYP2E1 induction by chronic ethanol feeding is a major contributor, producing high levels of ROS and lipid peroxidation byproducts that damage mitochondrial and cellular membranes, proteins, and DNA [20,21]. Acetaldehyde further amplifies oxidative stress by forming stable protein and DNA adducts, depleting GSH, impairing DNA repair, and promoting lipid peroxidation [8]. In addition, ethanol increases NO production through iNOS, which reacts with superoxide to generate peroxynitrite, a highly toxic RNS that exacerbates hepatocellular injury [20].

Chronic alcohol consumption also weakens antioxidant systems, including depletion of the mitochondrial GSH pool due to defective transport from the cytosol, and reduced activity of superoxide dismutase (SOD), catalase, and glutathione peroxidase (GPx), thereby diminishing ROS detoxification [21]. Ethanol-mediated changes in the NADH/NAD^+^ ratio disrupt cellular redox state and further enhance ROS production, while ROS act as signaling molecules activating redox-sensitive pathways such as nuclear factor-kappa B (NF-κB), c-Jun N-terminal kinase (JNK), and p38 mitogen-activated protein kinase (MAPK), promoting pro-inflammatory cytokine production and hepatocyte apoptosis [21]. Additionally, ethanol-induced mitochondrial damage leads to the release of proapoptotic factors and amplification of oxidative damage [24].

The interplay between oxidative stress and inflammation is central to ALD progression. Ethanol-induced ROS not only cause direct hepatocyte damage but also activate Kupffer cells, stimulating NF-κB–dependent transcription of TNFα, interleukin-1β (IL-1β), and other cytokines that recruit neutrophils and monocytes, which generate additional ROS/RNS [26]. This establishes a vicious cycle of oxidative stress and inflammation that accelerates hepatocyte death, fibrogenesis, and disease progression. In parallel, alcohol increases gut permeability, facilitating LPS translocation to the liver, where it activates Toll-like receptor 4 (TLR4) on Kupffer cells, hepatocytes, endothelial cells, and HSCs [29]. LPS/TLR4 signaling triggers NF-κB and interferon regulatory factor 3 (IRF3) activation [152], further amplified by ROS generated through nicotinamide adenine dinucleotide phosphate (NADPH) oxidase [153], thereby tightly linking endotoxemia, oxidative stress, and inflammation in ALD.

Reduced MAT1A expression and SAMe depletion further exacerbate these effects by compromising GSH-mediated detoxification [63], stabilizing ROS-generating enzymes such as CYP2E1 [81,86], and increasing TNF-α concentrations [154].

### 3.3. Mitochondrial Dysfunction

Mitochondria are critical regulators of hepatic energy metabolism, fatty acid oxidation, and redox homeostasis, making them particularly sensitive to alcohol-induced injury [23,24]. Chronic ethanol consumption causes profound mitochondrial dysfunction, including alterations in mitochondrial proteome (e.g., decreased levels of OXPHOS subunits such as NDUFS3 and COXIV, reduced β-oxidation enzymes including CPT1A, and impaired mitochondrial antioxidant defenses such as SOD2), as well as morphological abnormalities, impaired OXPHOS, reduced membrane potential, ATP depletion, mtDNA damage, excessive ROS generation, and decreased β-oxidation [23,24,97,104,155,156].

These changes disrupt hepatocellular energy balance, promote lipid accumulation, and sensitize hepatocytes to oxidative stress and apoptosis, thereby driving the progression of ALD. Ethanol exposure also causes structural abnormalities such as megamitochondria, loss of cristae, and reduced mitochondrial biogenesis [156,157]. In parallel, ethanol disrupts mitochondrial quality control processes such as biogenesis, dynamics, and mitophagy, further aggravating mitochondrial injury and contributing to hepatocyte damage, steatosis, inflammation, and fibrosis [24]. Moreover, alcohol increases mitochondrial Ca^2+^ levels and oxidative stress, while sensitizing liver mitochondria to mitochondrial permeability transition pore (mPTP) opening [158]. Collectively, these changes promote apoptosis by disrupting the balance between pro-apoptotic and anti-apoptotic factors in ALD.

Additional mechanisms include acetaldehyde toxicity, which induces protein and DNA adduct formation, depletes GSH and enhances lipid peroxidation [159,160]. Moreover, the increase in NADH/NAD^+^ ratio caused by ethanol suppresses fatty acid and glucose oxidation [161]. By elevating malonyl-CoA and inhibiting carnitine palmitoyltransferase I (CPT1) activity, ethanol restricts fatty acid transport into mitochondria, reducing β-oxidation while simultaneously stimulating de novo lipogenesis [22]. Ethanol further suppresses 5′ AMP-activated protein kinase (AMPK) signaling and downregulates sirtuin pathways (notably SIRT1 and SIRT3), leading to hyperacetylation of mitochondrial proteins, impaired oxidative metabolism, and defective antioxidant defenses [162,163,164].

MATα1 and SAMe deficiency exacerbate these mitochondrial defects. Loss of SAMe disrupts methylation-dependent processes within mitochondria, including ETC assembly, and the generation of ATP and ROS [67,81,104,105,158]. Ethanol-fed rats exhibit reduced activities of liver mitochondrial respiratory chain complexes I and IV, an effect that is prevented by SAMe administration [62]. Decreased SAMe also impairs methylation of mitochondrial proteins such as CYP2E1 [81] and MCJ [150] in mouse liver, enhancing their stability, thereby increasing ROS-generating capacity and impairing mitochondrial respiration. Furthermore, SAMe deficiency reduces PC synthesis via the PEMT pathway, compromising membrane integrity [106,165]. In line with these findings, more recent studies in hepatocytes and mice have shown that ethanol-induced mitochondrial MATα1 loss impairs mitochondrial methylation capacity, membrane potential, and respiration, thereby increasing oxidative stress, hepatic steatosis, and hepatocellular injury [89,104].

MATα1 and SAMe play a central role in preserving mitochondrial integrity, and their deficiency markedly contributes to mitochondrial dysfunction, a key driver of alcohol-induced liver injury.

### 3.4. Other Factors

Beyond aberrant methylation, oxidative stress, and mitochondrial dysfunction, loss of MAT1A and depletion of SAMe contribute to ALD progression through multiple additional molecular pathways.

One major mechanism involves dysregulation of sphingolipid metabolism via acid sphingomyelinase (ASMase). SAMe deficiency has been shown to increase ASMase activity, leading to elevated ceramide levels in hepatocytes [95]. Ceramides act as a bioactive lipid that triggers ER stress, disrupts Ca^2+^ homeostasis, and sensitizes hepatocytes to apoptosis, particularly in the context of ethanol consumption. Moreover, ceramide can promote activation of HSCs, contributing to fibrogenesis and the progression from steatosis to fibrosis [166].

Reduced SAMe availability also triggers inflammatory and cell death pathways by sensitizing hepatocytes to TNFα–mediated apoptosis, enhancing JNK and p38 MAPK signaling, further promoting hepatocellular injury [167,168]. These effects are compounded by altered gut-liver crosstalk, as SAMe depletion amplifies the hepatic response to gut-derived endotoxins, enhancing inflammatory signaling and cytokine release [82,154].

Beyond methylation, SAMe deficiency has been associated with altered post-translational modifications, including phosphorylation and SUMOylation. Alcohol disrupts these pathways, contributing to impaired signaling, protein stability, and stress responses. Although the direct involvement of SAMe in these processes has not been fully defined, its central role in hepatic metabolism and redox regulation suggests it may influence these alterations.

Collectively, these findings highlight that SAMe and MATα1 regulate multiple interconnected molecular pathways—including ASMase/ceramide accumulation, ER stress, apoptotic signaling, and inflammatory responses—that likely converge to drive ALD pathogenesis. The broad influence of SAMe/MAT1A underscores their central role as integrators of hepatocellular homeostasis and suggests that their depletion can simultaneously dysregulate numerous protective mechanisms, amplifying liver injury. Understanding the diverse pathways controlled by SAMe/MAT1A is, therefore, critical, as it provides insight into how methionine metabolism impacts ALD progression and identifies potential multifaceted therapeutic targets to mitigate disease development.

## 4. Therapeutic Potential of Targeting MAT1A and SAMe in ALD

Restoring SAMe levels and MATα1 function offers a promising therapeutic approach in ALD, as these interventions target key pathways underlying liver injury, from redox imbalance to inflammation and cell death. This section summarizes current evidence supporting strategies that modulate the MAT1A–SAMe axis to prevent or attenuate alcohol-induced liver damage.

### 4.1. Preclinical Evidence: Studies on the Protective Effect of SAMe and MAT1A in ALD

Preclinical studies in cell and animal models provide strong evidence that SAMe and MATα1 protect against alcohol-induced liver injury. In primary hepatocytes, SAMe treatment prevents ethanol-induced depletion of mitochondrial GSH and preserves mitochondrial function [63]. Similarly, in isolated perfused rat liver, SAMe attenuated ethanol-induced hepatotoxicity, as evidenced by decreased release of aspartate transaminase (AST) and lactate dehydrogenase, restoration of mitochondrial and total GSH, and preservation of mitochondrial respiration [169].

In vivo studies further support these findings. In ethanol-fed rats, both preventive and recovery SAMe treatment completely prevented hepatic triglyceride accumulation and steatosis while restoring hepatic SAMe and GSH levels [64]. Long-term studies in baboons demonstrated that SAMe supplementation corrected ethanol-induced depletion of hepatic SAMe and GSH, reduced giant mitochondria formation, and lowered plasma AST levels, consistent with reduced liver injury [58]. In rats, SAMe restored mitochondrial and cytosolic GSH levels in the liver [63], prevented ethanol-induced increases in superoxide production, and inhibited iNOS induction, reducing both ROS and RNS accumulation in the liver [62]. Interestingly, SAMe—but not N-acetylcysteine (NAC)—restored the mitochondrial GSH pool in isolated hepatic mitochondria, highlighting its unique ability to protect mitochondrial redox status [65].

SAMe has also been tested in acute alcohol-induced liver injury. In mice exposed to three ethanol doses over 12 h, pretreatment with SAMe for three days reduced liver injury, preserved hepatic SAMe, restored mitochondrial GSH, mitigated mitochondrial permeability transition, and limited lipid peroxidation [66].

In rats fed ethanol for ≥31 days, 5 weeks, SAMe supplementation consistently demonstrated mitochondrial protective effects and reduced liver injury [62,97]. Specifically, Bailey and colleagues showed that in ethanol-fed rats, SAMe treatment prevented alcohol-induced mtDNA damage in the liver while preserving mitochondrial respiration and membrane permeability in isolated hepatic mitochondria [62,67]. In the same experiments, SAMe also mitigated iNOS induction and normalized sensitivity to Ca^2+^-induced permeability transition and NO-mediated inhibition of respiration [62,67]. Hepatic hypoxia was also alleviated, collectively preserving mitochondrial bioenergetics [62,67]. Proteomic studies further showed that SAMe co-administration in ethanol-fed rats maintained the abundance of key mitochondrial proteins—including OXPHOS subunits, chaperones, β-oxidation enzymes, sulfur metabolism proteins, and dehydrogenases involved in methionine, glycine, and choline metabolism—as well as preserved Complex I and IV activities [62,97].

In ethanol-fed micropigs on a folate-deficient diet, SAMe supplementation attenuated oxidative liver injury by reducing markers of oxidative stress (malondialdehyde, hydroxynonenal, nitrotyrosine), restoring hepatic SAMe and GSH levels, and decreasing CYP2E1 and iNOS activity [68]. In the same model, SAMe also reduced hepatic lipid synthesis and steatosis by maintaining adiponectin-AMPK signaling and preventing upregulation of SREBP-1c and lipogenic enzymes [133]. Furthermore, in ethanol-fed rats, SAMe administration prevented the upregulation of TLR signaling [170] in the liver, a key mediator of the proinflammatory response in ALD [8].

Recent studies targeting mitochondrial MATα1 directly have shown similarly promising results. In hepatocytes, expression of a stable MATα1 mutant (S114A) protected against ethanol-induced mitochondrial injury, oxidative stress, and lipid accumulation [104]. In line with this, in ethanol-fed mice on the NIAAA diet, CRISPR-mediated stabilization of mitochondrial MATα1 (K48) preserved hepatic SAMe levels, reduced liver injury (lower serum levels of alanine and aspartate aminotransferases), and decreased steatosis [89]. Higher mitochondrial MATα1 content correlated with increased methylation and expression of proteins involved in major mitochondrial metabolic pathways [104].

Betaine supplementation has been shown in multiple preclinical studies to restore the hepatic SAMe/SAH ratio, increase SAMe levels, and improve methylation capacity in the livers of alcohol-fed animals. These effects were accompanied by attenuation of alcohol-induced liver injury, including reductions in steatosis, oxidative stress, mitochondrial dysfunction, and inflammatory signaling, measured in liver tissues. By supporting the BHMT-mediated remethylation of homocysteine to methionine, betaine effectively replenishes SAMe and protects hepatocytes from ethanol-induced damage, highlighting its potential as a complementary therapeutic strategy in ALD [56,59,137,171,172,173,174].

Together, these preclinical studies demonstrate that MAT1A deficiency and SAMe depletion exacerbate ALD, while interventions that restore SAMe levels or preserve MATα1 confer robust protection against liver injury (Table 3). This body of work provides strong support for targeting the MAT1A–SAMe axis as a therapeutic strategy in ALD.

### 4.2. Clinical Studies

Only a few randomized, placebo-controlled trials have evaluated the therapeutic potential of SAMe in patients with ALD, and results were mixed.

An early clinical study demonstrated that oral administration of 1.2 g/day SAMe for six months significantly increased hepatic GSH levels in patients with liver disease, including a small subgroup with ALD, providing initial evidence that SAMe can restore antioxidant capacity in humans [175]. Building on this, a larger multicenter, double-blind trial enrolled 123 patients with biopsy-confirmed alcoholic cirrhosis who were randomized to receive SAMe (1.2 g/day) or placebo for 24 months [69]. Although no significant difference was observed in the overall cohort, in a post hoc analysis SAMe treatment significantly improved transplant-free survival in patients with less-advanced cirrhosis, suggesting potential benefit when administered at earlier stages of disease. Since significance was only found in the post hoc analysis, the study remains to be confirmed. In contrast, in a more recent, smaller, shorter (24-week) randomized, double-blind, placebo-controlled trial, 37 abstinent patients with ALD received oral SAMe 1.2 g/day or placebo. SAMe increased circulating SAMe levels, but there were no significant differences between groups in liver enzymes, histology (performed in 8 placebo and 6 SAMe groups), or clinical outcomes [176]. However, the last study suffered from a small number of subjects enrolled in part due to a high dropout rate (~30% due to active drinking as abstinence was required to stay in the study) and a short treatment period [176], which limited the impact of the study. To better define the role of SAMe supplementation (1.2 g/day in two divided doses for 2 years) in 176 patients with Child Class A-B alcoholic cirrhosis, an NIAAA-funded multi-center, randomized, placebo-controlled clinical trial (NCT04250259) was launched in late 2020 and is anticipated to conclude in 2026. The trial is anticipated to address whether patients with Child Class A-B alcoholic cirrhosis benefit from SAMe supplementation, if there are biomarkers that predict response, and if SAMe impacts pathogenetic factors known to be important in ALD. It should be noted that not all cirrhotic patients may exhibit reduced hepatic SAMe levels, as suggested by the recent identification of MASLD subtypes [94], highlighting the potential for a personalized medicine approach in selecting patients most likely to benefit from SAMe therapy.

Taken together, these clinical studies show that SAMe consistently increases circulating and hepatic SAMe and GSH concentrations, but improvements in clinical outcomes remain to be confirmed in a large multi-center, randomized, placebo-controlled clinical trial that is underway.

### 4.3. Limitations and Future Strategies

Although preclinical models provide strong mechanistic evidence, SAMe supplementation and MAT1A preservation have yet to be successfully translated into effective therapies for ALD. Animal studies consistently show that SAMe protects against alcohol-induced oxidative stress, steatosis, and mitochondrial dysfunction, but these models cannot fully capture the chronicity, heterogeneity, and comorbidities of human disease. Differences in experimental design, including ethanol exposure, SAMe dosing, and timing of administration, further complicate extrapolation to patients. One additional limitation is that not all patients with ALD may be SAMe deficient, which could influence responsiveness to supplementation and highlights the need to identify patient subgroups that may benefit most.

Clinical trials have so far produced mixed results, underscoring the challenges of moving from bench to bedside, particularly in this patient population with high recidivism. Moreover, the poor and variable bioavailability of conventional SAMe formulations likely limits therapeutic benefit, particularly with respect to mitochondrial delivery, where SAMe plays a critical role in maintaining redox balance and methylation capacity. Addressing these limitations will require new strategies. Improved formulations, including mitochondria-targeted SAMe, may enhance uptake and therapeutic efficacy. Beyond direct supplementation, approaches that boost endogenous MAT1A expression or target epigenetic regulation of MAT1A could restore SAMe homeostasis more effectively. Combination therapies—for example, pairing SAMe with betaine, folate, or vitamin B_6_—may also prove beneficial by supporting complementary pathways. Finally, biomarker-driven patient selection and well-designed, adequately powered clinical trials will be essential to determine whether the robust benefits observed in experimental models can be replicated in humans.

## 5. Perspectives and Conclusions

In conclusion, the MAT1A–SAMe is a central regulator of liver homeostasis in ALD. Preclinical evidence highlights its role in mitigating oxidative stress, preserving mitochondrial function, and limiting hepatocellular damage, while early clinical studies suggest potential therapeutic benefits. Future research aimed at optimizing SAMe delivery, preserving MAT1A activity, particularly within the mitochondria, and exploring combinatorial approaches holds promise for translating these findings into effective interventions. Overall, targeting the MAT1A–SAMe pathway represents a compelling strategy to prevent and treat alcoholic liver disease.

## Figures and Tables

**Table 1:** SAMe Levels in experimental and clinical ALD.

Study Type	Model	SAMe Level Change	Reference
Animal (rat)	Low-protein + 5 % ethanol diet (28 days)	Hepatic SAMe: 46 % ↓ in ALD (absolute values NR)	[98]
Animal (rat)	Intragastric ethanol + high-fat diet (9 weeks)	Hepatic SAMe (nmol/mg protein): (40 % ↓ in ALD) Control: 0.43 ± 0.03 Ethanol: 0.27 ± 0.02 *	[55]
Animal (rat)	Ethanol diet (28 days)	Hepatic SAMe (nmol/liver): (21 % ↓ in ALD) Control: 675 Ethanol: 533 *	[99]
Animal (rat)	Ethanol diet (21 days)	Hepatic SAMe (nmol/g): (40 % ↓ in ALD) Control: 100.9 ± 13.3 Ethanol: 59.6 ± 10.7 *	[57]
Animal (Baboon)	Ethanol diet (18–36 months)	Hepatic SAMe (nmol/g liver): (26 % ↓ in ALD) Control: 108.90 ± 8.20 Ethanol: 74.60 ± 2.40 *	[58]
Animal (rat)	Ethanol diet (31 days or 5 weeks)	Hepatic SAMe (nmol/g liver/g body weight): Control: 2.09 ± 0.17 Ethanol: 1.62 ± 0.13 *	[62,67,97]
Animal (mouse)	Acute ethanol gavage (5 g/kg every 12 h × 3 doses)	Hepatic SAMe (nmol/mg protein): Control: 0.621 ± 0.051 Ethanol: 0.180 ± 0.016 *	[66]
Animal (rat)	Ethanol diet + polyenylphosphatidylcholine (2 months)	Hepatic SAMe (nmol/g): (47 % ↓ in ALD) Control: 68.2 ± 5.1 Ethanol: 36.2 ± 3.4 *	[100]
Animal (perfused rat liver)	Isolated perfused liver from ethanol-fed mice	Hepatic SAMe (nmol/g): (≈42 % ↓ in ALD) Control: 176.7 ± 22.1 Ethanol: 73.4 ± 7.0 *	[101]
Animal (mouse)	Ethanol feeding (C57BL/6, 4 weeks)	↓ cytosolic & mitochondrial SAMe (>40 % ↓; absolute values NR)	[102]
Human	Alcoholic hepatitis (biopsy, *n* = 6)	Plasma SAMe (µM): (≈50 % ↓ in ALD) Control: 23.6 ± 7 Ethanol: 12.5± 11 *	[60]

NR = not reported, * represents significant vs. control. ↓ represents reduction in levels.

**Table 2:** Regulatory mechanisms of MAT1A in healthy and ALD liver.

Regulation of MAT1A	Mechanism of MAT1 Regulation in the Liver
Transcriptional regulation	- Promoter hypermethylation (−) [111] - Promoter hyperacetylation (+) [111] - Glucocorticoids (+) [109] - C/EBP (+) [109,110] - HNF (+) [109] - AP1 (+) [109] - c-MYC (−) [112,113] - MAX (−) [112,113] - MAFG (−) [112] - c-MAF (−) [112,113]
Post-transcriptional regulation	- AUF1 (−) [114] - miR-22 (−) [115] - mir-29b (−) [115] - miR-485-3p (−) [116] - miR-495 (−) [116] - miR-664 (−) [116]
Post-translational regulation	- Oxidation: C121 (−) [118] - Nitrosylation: C121) (−) [120] - Phosphorylation: Ser114 (−) [104], S180 (−) [122], T202 (−) [122], T342 (−) [121] - SUMOylation: K48 (−) [89]

Red color indicates the mechanism in ALD.

**Table 3:** Summary of SAMe/MAT1A Interventions Across In Vitro, Animal, and Human Models of ALD.

Study Type	Species/Sex/Age	SAMe Dose & Route/MAT1A Mutation	Regimen	SAMe Levels Reached (Reported)	Main Outcomes (↑ Increase, ↓ Decrease)	Ref
In vitro/In vivo	Primary rat hepatocytes (male Sprague Dawley)	0.4 mg/mL SAMe in liquid diet (1 µmol/mL; 40–50 mL diet/day)	4 weeks	NR	↓ mitochondrial GSH depletion, ↑ mitochondrial function	[63,65]
In vitro/In vivo	Primary rat hepatocytes (male Wistar)	25 mg/kg SAMe IM, 3×/day	5 days	NR	↓ AST, ↓ LDH release, ↑ mitochondrial GSH, ↑ mitochondrial respiration	[169]
In vivo	Female Wistar rats	25 mg/kg intramuscular injection thrice a day	16 days	NR	↓ hepatic TG, ↑ hepatic GSH, ↓ blood and liver acetaldehyde	[64]
In vivo	Baboon (sex NR); 4–6 years	SAMe in diet: average 26.4 ± 1.4 mg/kg/day	18–36 months	Hepatic SAMe (nmol/g liver): Control: 108.90 ± 8.20 Ethanol: 74.60 ± 2.40 * SAMe + Ethanol: **102.10 ± 15.40** ^†^	↑ hepatic SAMe & GSH, ↓ AST, ↓ glutamic dehydrogenase ↓ no. of giant mitochondria ↓ liver injury	[58]
In vivo	Male Sprague Dawley rats	0.8 mg active SAMe/mL in diet	31 days or 5 weeks	Hepatic SAMe (nmol/g liver/g body weight): Control: 2.09 ± 0.17 Ethanol: 1.62 ± 0.13 * SAMe + Ethanol: **1.95 ± 0.09 ^†^** Mitochondrial SAMe (nmol/mg protein): Control: 0.037 ± 0.002 Ethanol: 0.057 ± 0.005 SAMe + Ethanol: **0.082 ± 0.004 ^†^**	↓ steatosis ↓ hypoxia, ↓ mitochondrial injury, ↓ iNOS, ROS, NO accumulation ↑ mitochondrial function	[62,67,97]
In vivo	Male C57BL/6 mice	50 mg/kg SAMe i.p. every 12 h × 3 doses	SAMe given before 5 g/kg ethanol gavage (every 12 h × 3 doses)	Hepatic SAMe (nmol/mg protein): Control: 0.621 ± 0.051 Ethanol: 0.180 ± 0.016 * SAMe + Ethanol: **13.606 ± 8.768 ^†^**	↓ ALT, ↓ lipid peroxidation, ↑ mitochondrial GSH, ↓ steatosis	[66]
In vivo	Yucatan micropigs, 6-month-old	0.4 g/1000 kcal in diet (90 kcal/kg/day)	14 weeks	No significant hepatic SAMe change (absolute values NR)	↓ steatosis, ↑ GSH, ↑ MnSOD, ↓ Nitrotyrosine, ↓ GSSG, NADPH oxidase, iNOS ↓ CYP2E1	[68]
In vivo	Female C57BL/6 mice, 3-month-old	CRISPR/Cas9 gene editing of MATα1 at K48	3 doses (days 1, 5, and 9) during the NIAAA model (11 days)	Hepatic SAMe (nmol/L/µg protein): WT Control: ~7.5 WT Ethanol: ~5 * HDR Control: ~7.5 HDR Ethanol: ~11.5**^ †^**	↓ steatosis ↓ hepatic TGs ↑ mito function, ↑ ATP ↓ mROS ↓ ALT, AST	[89]
Human	123 patients; 106 men, 17 women (Child A–C)	1200 mg/day SAMe (oral)	RCT; double-blind; 2 years	NR	No overall survival benefit, ↑ overall survival/transplant-free survival in Child A–B subgroup (after excluding Child C group)	[69]
Human	32 patients (8 normal control, 8 ALD, 9 alcoholic cirrhosis, 9 ALD + SAMe); mean age 44	1200 mg/day SAMe (oral)	Placebo-controlled; 6 months	NR	↑ hepatic GSH vs. placebo	[175]
Human	Adults, both sexes	1200 mg/day SAMe (oral)	RCT; double-blind; 24 weeks	↑ fasting serum SAMe (absolute values NR)	No significant improvement in liver biochemical tests	[176]

NR = not reported, * represents significant vs. control; ^†^ represents significant vs. ethanol. ↑ represents increase and ↓ represents decrease in levels.

## Data Availability

No new data were created or analyzed in this study. Data sharing is not applicable to this article.

## References

[1] Sengupta S., Gill V., Mellinger J.L. (2024). Alcohol-Associated Liver Disease and Public Health Policies. Hepatology.

[2] Huang D.Q., Terrault N.A., Tacke F., Gluud L.L., Arrese M., Bugianesi E., Loomba R. (2023). Global Epidemiology of Cirrhosis–Aetiology, Trends and Predictions. Nat. Rev. Gastroenterol. Hepatol..

[3] Mackowiak B., Fu Y., Maccioni L., Gao B. (2024). Alcohol-Associated Liver Disease. J. Clin. Investig..

[4] Jun S., Park H., Kim U.-J., Choi E.J., Lee H.A., Park B., Lee S.Y., Jee S.H., Park H. (2023). Cancer Risk Based on Alcohol Consumption Levels: A Comprehensive Systematic Review and Meta-Analysis. Epidemiol. Health.

[5] Cecchini M., Filippini T., Whelton P.K., Iamandii I., Di Federico S., Boriani G., Vinceti M. (2024). Alcohol Intake and Risk of Hypertension: A Systematic Review and Dose-Response Meta-Analysis of Nonexperimental Cohort Studies. Hypertension.

[6] Åberg F., Jiang Z.G., Cortez-Pinto H., Männistö V. (2024). Alcohol-Associated Liver Disease-Global Epidemiology. Hepatology.

[7] Bataller R., Arab J.P., Shah V.H. (2022). Alcohol-Associated Hepatitis. N. Engl. J. Med..

[8] Wu X., Fan X., Miyata T., Kim A., Cajigas-Du Ross C.K., Ray S., Huang E., Taiwo M., Arya R., Wu J. (2023). Recent Advances in Understanding of Pathogenesis of Alcohol-Associated Liver Disease. Annu. Rev. Pathol..

[9] Mullish B.H., Thursz M.R. (2024). Alcohol-Associated Liver Disease: Emerging Therapeutic Strategies. Hepatology.

[10] Moreno C., Langlet P., Hittelet A., Lasser L., Degré D., Evrard S., Colle I., Lemmers A., Devière J., Moine O.L. (2010). Enteral Nutrition with or without N-Acetylcysteine in the Treatment of Severe Acute Alcoholic Hepatitis: A Randomized Multicenter Controlled Trial. J. Hepatol..

[11] Higuera-de la Tijera F., Servín-Caamaño A.I., Cruz-Herrera J., Serralde-Zúñiga A.E., Abdo-Francis J.M., Gutiérrez-Reyes G., Pérez-Hernández J.L. (2014). Treatment with Metadoxine and Its Impact on Early Mortality in Patients with Severe Alcoholic Hepatitis. Ann. Hepatol..

[12] Marot A., Singal A.K., Moreno C., Deltenre P. (2020). Granulocyte Colony-Stimulating Factor for Alcoholic Hepatitis: A Systematic Review and Meta-Analysis of Randomised Controlled Trials. JHEP Rep..

[13] Arab J.P., Sehrawat T.S., Simonetto D.A., Verma V.K., Feng D., Tang T., Dreyer K., Yan X., Daley W.L., Sanyal A. (2020). An Open-Label, Dose-Escalation Study to Assess the Safety and Efficacy of IL-22 Agonist F-652 in Patients With Alcohol-Associated Hepatitis. Hepatology.

[14] Ki S.H., Park O., Zheng M., Morales-Ibanez O., Kolls J.K., Bataller R., Gao B. (2010). Interleukin-22 Treatment Ameliorates Alcoholic Liver Injury in a Murine Model of Chronic-Binge Ethanol Feeding: Role of Signal Transducer and Activator of Transcription 3. Hepatology.

[15] Naveau S., Chollet-Martin S., Dharancy S., Mathurin P., Jouet P., Piquet M.-A., Davion T., Oberti F., Broët P., Emilie D. (2004). A Double-Blind Randomized Controlled Trial of Infliximab Associated with Prednisolone in Acute Alcoholic Hepatitis. Hepatology.

[16] Boetticher N.C., Peine C.J., Kwo P., Abrams G.A., Patel T., Aqel B., Boardman L., Gores G.J., Harmsen W.S., McClain C.J. (2008). A Randomized, Double-Blinded, Placebo-Controlled Multicenter Trial of Etanercept in the Treatment of Alcoholic Hepatitis. Gastroenterology.

[17] Han S.H., Suk K.T., Kim D.J., Kim M.Y., Baik S.K., Kim Y.D., Cheon G.J., Choi D.H., Ham Y.L., Shin D.H. (2015). Effects of Probiotics (Cultured Lactobacillus Subtilis/Streptococcus Faecium) in the Treatment of Alcoholic Hepatitis: Randomized-Controlled Multicenter Study. Eur. J. Gastroenterol. Hepatol..

[18] Philips C.A., Phadke N., Ganesan K., Ranade S., Augustine P. (2018). Corticosteroids, Nutrition, Pentoxifylline, or Fecal Microbiota Transplantation for Severe Alcoholic Hepatitis. Indian. J. Gastroenterol..

[19] Philips C.A., Pande A., Shasthry S.M., Jamwal K.D., Khillan V., Chandel S.S., Kumar G., Sharma M.K., Maiwall R., Jindal A. (2017). Healthy Donor Fecal Microbiota Transplantation in Steroid-Ineligible Severe Alcoholic Hepatitis: A Pilot Study. Clin. Gastroenterol. Hepatol..

[20] Michalak A., Lach T., Cichoż-Lach H. (2021). Oxidative Stress-A Key Player in the Course of Alcohol-Related Liver Disease. J. Clin. Med..

[21] Tan H.K., Yates E., Lilly K., Dhanda A.D. (2020). Oxidative Stress in Alcohol-Related Liver Disease. World J. Hepatol..

[22] You M., Arteel G.E. (2019). Effect of Ethanol on Lipid Metabolism. J. Hepatol..

[23] Nassir F., Ibdah J.A. (2014). Role of Mitochondria in Alcoholic Liver Disease. World J. Gastroenterol..

[24] Thoudam T., Gao H., Jiang Y., Huda N., Yang Z., Ma J., Liangpunsakul S. (2024). Mitochondrial Quality Control in Alcohol-Associated Liver Disease. Hepatol. Commun..

[25] Mandrekar P., Mandal A. (2024). Pathogenesis of Alcohol-Associated Liver Disease. Clin. Liver Dis..

[26] Yang T., Gu Z., Feng J., Shan J., Qian C., Zhuang N. (2025). Non-Parenchymal Cells: Key Targets for Modulating Chronic Liver Diseases. Front. Immunol..

[27] Mandrekar P., Szabo G. (2009). Signalling Pathways in Alcohol-Induced Liver Inflammation. J. Hepatol..

[28] Chen P., Stärkel P., Turner J.R., Ho S.B., Schnabl B. (2015). Dysbiosis-Induced Intestinal Inflammation Activates TNFRI and Mediates Alcoholic Liver Disease in Mice. Hepatology.

[29] Szabo G., Bala S. (2010). Alcoholic Liver Disease and the Gut-Liver Axis. World J. Gastroenterol..

[30] Wang Z.-G., Dou X.-B., Zhou Z.-X., Song Z.-Y. (2016). Adipose Tissue-Liver Axis in Alcoholic Liver Disease. World J. Gastrointest. Pathophysiol..

[31] Patidar P., Hirani N., Bharti S., Baig M.S. (2024). Key Regulators of Hepatic Stellate Cell Activation in Alcohol Liver Disease: A Comprehensive Review. Int. Immunopharmacol..

[32] Ramos-Tovar E., Muriel P. (2020). Molecular Mechanisms That Link Oxidative Stress, Inflammation, and Fibrosis in the Liver. Antioxidants.

[33] Zeng G., Gao H., Jiang Y., Huda N., Thoudam T., Yang Z., Ma J., Sun J., Liangpunsakul S. (2025). Non-Coding RNAs in Alcohol-Associated Liver Disease. Liver Res..

[34] Habash N.W., Sehrawat T.S., Shah V.H., Cao S. (2022). Epigenetics of Alcohol-Related Liver Diseases. JHEP Rep..

[35] Mukherji A., Bailey S.M., Staels B., Baumert T.F. (2019). The Circadian Clock and Liver Function in Health and Disease. J. Hepatol..

[36] Daniels L.J., Kay D., Marjot T., Hodson L., Ray D.W. (2023). Circadian Regulation of Liver Metabolism: Experimental Approaches in Human, Rodent, and Cellular Models. Am. J. Physiol. Cell Physiol..

[37] Summa K.C., Voigt R.M., Forsyth C.B., Shaikh M., Cavanaugh K., Tang Y., Vitaterna M.H., Song S., Turek F.W., Keshavarzian A. (2013). Disruption of the Circadian Clock in Mice Increases Intestinal Permeability and Promotes Alcohol-Induced Hepatic Pathology and Inflammation. PLoS ONE.

[38] Bailey S.M. (2018). Emerging Role of Circadian Clock Disruption in Alcohol-Induced Liver Disease. Am. J. Physiol. Gastrointest. Liver Physiol..

[39] Lu S.C., Mato J.M. (2012). S-Adenosylmethionine in Liver Health, Injury, and Cancer. Physiol. Rev..

[40] Brosnan J.T., Brosnan M.E., Bertolo R.F.P., Brunton J.A. (2007). Methionine: A Metabolically Unique Amino Acid. Livest. Sci..

[41] Murray B., Barbier-Torres L., Fan W., Mato J.M., Lu S.C. (2019). Methionine Adenosyltransferases in Liver Cancer. World J. Gastroenterol..

[42] Finkelstein J.D. (2007). Metabolic Regulatory Properties of S-Adenosylmethionine and S-Adenosylhomocysteine. Clin. Chem. Lab. Med..

[43] Cook R.J., Wagner C. (1984). Glycine N-Methyltransferase Is a Folate Binding Protein of Rat Liver Cytosol. Proc. Natl. Acad. Sci. USA.

[44] Ogawa H., Fujioka M. (1982). Purification and Properties of Glycine N-Methyltransferase from Rat Liver. J. Biol. Chem..

[45] Takusagawa F., Ogawa H., Fujioka M. (1999). Glycine N-Methyltransferase, a Tetrameric Enzyme. S-Adenosylmethionine-Dependent Methyltransferases.

[46] Augoustides-Savvopoulou P., Luka Z., Karyda S., Stabler S.P., Allen R.H., Patsiaoura K., Wagner C., Mudd S.H. (2003). Glycine N-Methyltransferase Deficiency: A New Patient with a Novel Mutation. J. Inherit. Metab. Dis..

[47] Luka Z., Mudd S.H., Wagner C. (2009). Glycine N-Methyltransferase and Regulation of S-Adenosylmethionine Levels. J. Biol. Chem..

[48] Luka Z., Capdevila A., Mato J.M., Wagner C. (2006). A Glycine N-Methyltransferase Knockout Mouse Model for Humans with Deficiency of This Enzyme. Transgenic Res..

[49] Liu S.-P., Li Y.-S., Chen Y.-J., Chiang E.-P., Li A.F.-Y., Lee Y.-H., Tsai T.-F., Hsiao M., Hwang S.-F., Chen Y.-M.A. (2007). Glycine N-Methyltransferase−/− Mice Develop Chronic Hepatitis and Glycogen Storage Disease in the Liver. Hepatology.

[50] Martínez-Chantar M.L., Vázquez-Chantada M., Ariz U., Martínez N., Varela M., Luka Z., Capdevila A., Rodríguez J., Aransay A.M., Matthiesen R. (2008). Loss of the Glycine N-Methyltransferase Gene Leads to Steatosis and Hepatocellular Carcinoma in Mice. Hepatology.

[51] Lin M., Wang J., Chai Y., Chen X., Zhao D., Xie Z., Jiang J., Li H., Huang L., Xing S. (2025). Homocysitaconate Controls Inflammation through Reshaping Methionine Metabolism and N-Homocysteinylation. Cell Metab..

[52] Lu S.C. (1998). Methionine Adenosyltransferase and Liver Disease: It’s All about SAM. Gastroenterology.

[53] Mato J.M., Alvarez L., Ortiz P., Pajares M.A. (1997). S-Adenosylmethionine Synthesis: Molecular Mechanisms and Clinical Implications. Pharmacol. Ther..

[54] Horowitz J.H., Rypins E.B., Henderson J.M., Heymsfield S.B., Moffitt S.D., Bain R.P., Chawla R.K., Bleier J.C., Rudman D. (1981). Evidence for Impairment of Transsulfuration Pathway in Cirrhosis. Gastroenterology.

[55] Lu S.C., Huang Z.Z., Yang H., Mato J.M., Avila M.A., Tsukamoto H. (2000). Changes in Methionine Adenosyltransferase and S-Adenosylmethionine Homeostasis in Alcoholic Rat Liver. Am. J. Physiol. Gastrointest. Liver Physiol..

[56] Barak A.J., Beckenhauer H.C., Junnila M., Tuma D.J. (1993). Dietary Betaine Promotes Generation of Hepatic S-Adenosylmethionine and Protects the Liver from Ethanol-Induced Fatty Infiltration. Alcohol. Clin. Exp. Res..

[57] Trimble K.C., Molloy A.M., Scott J.M., Weir D.G. (1993). The Effect of Ethanol on One-Carbon Metabolism: Increased Methionine Catabolism and Lipotrope Methyl-Group Wastage. Hepatology.

[58] Lieber C.S., Casini A., DeCarli L.M., Kim C.I., Lowe N., Sasaki R., Leo M.A. (1990). S-Adenosyl-L-Methionine Attenuates Alcohol-Induced Liver Injury in the Baboon. Hepatology.

[59] Kharbanda K.K., Mailliard M.E., Baldwin C.R., Beckenhauer H.C., Sorrell M.F., Tuma D.J. (2007). Betaine Attenuates Alcoholic Steatosis by Restoring Phosphatidylcholine Generation via the Phosphatidylethanolamine Methyltransferase Pathway. J. Hepatol..

[60] Lee T.D., Sadda M.R., Mendler M.H., Bottiglieri T., Kanel G., Mato J.M., Lu S.C. (2004). Abnormal Hepatic Methionine and Glutathione Metabolism in Patients with Alcoholic Hepatitis. Alcohol. Clin. Exp. Res..

[61] Kamimura S., Gaal K., Britton R.S., Bacon B.R., Triadafilopoulos G., Tsukamoto H. (1992). Increased 4-Hydroxynonenal Levels in Experimental Alcoholic Liver Disease: Association of Lipid Peroxidation with Liver Fibrogenesis. Hepatology.

[62] King A.L., Mantena S.K., Andringa K.K., Millender-Swain T., Dunham-Snary K.J., Oliva C.R., Griguer C.E., Bailey S.M. (2016). The Methyl Donor S-Adenosylmethionine Prevents Liver Hypoxia and Dysregulation of Mitochondrial Bioenergetic Function in a Rat Model of Alcohol-Induced Fatty Liver Disease. Redox Biol..

[63] García-Ruiz C., Morales A., Colell A., Ballesta A., Rodés J., Kaplowitz N., Fernández-Checa J.C. (1995). Feeding S-Adenosyl-L-Methionine Attenuates Both Ethanol-Induced Depletion of Mitochondrial Glutathione and Mitochondrial Dysfunction in Periportal and Perivenous Rat Hepatocytes. Hepatology.

[64] Feo F., Pascale R., Garcea R., Daino L., Pirisi L., Frassetto S., Ruggiu M.E., Di Padova C., Stramentinoli G. (1986). Effect of the Variations of S-Adenosyl-L-Methionine Liver Content on Fat Accumulation and Ethanol Metabolism in Ethanol-Intoxicated Rats. Toxicol. Appl. Pharmacol..

[65] Colell A., García-Ruiz C., Morales A., Ballesta A., Ookhtens M., Rodés J., Kaplowitz N., Fernández-Checa J.C. (1997). Transport of Reduced Glutathione in Hepatic Mitochondria and Mitoplasts from Ethanol-Treated Rats: Effect of Membrane Physical Properties and S-Adenosyl-L-Methionine. Hepatology.

[66] Song Z., Zhou Z., Chen T., Hill D., Kang J., Barve S., McClain C. (2003). S-Adenosylmethionine (SAMe) Protects against Acute Alcohol Induced Hepatotoxicity in Mice. J. Nutr. Biochem..

[67] Bailey S.M., Robinson G., Pinner A., Chamlee L., Ulasova E., Pompilius M., Page G.P., Chhieng D., Jhala N., Landar A. (2006). S-Adenosylmethionine Prevents Chronic Alcohol-Induced Mitochondrial Dysfunction in the Rat Liver. Am. J. Physiol. Gastrointest. Liver Physiol..

[68] Villanueva J.A., Esfandiari F., White M.E., Devaraj S., French S.W., Halsted C.H. (2007). S-Adenosylmethionine Attenuates Oxidative Liver Injury in Micropigs Fed Ethanol with a Folate-Deficient Diet. Alcohol. Clin. Exp. Res..

[69] Mato J.M., Cámara J., Fernández de Paz J., Caballería L., Coll S., Caballero A., García-Buey L., Beltrán J., Benita V., Caballería J. (1999). S-Adenosylmethionine in Alcoholic Liver Cirrhosis: A Randomized, Placebo-Controlled, Double-Blind, Multicenter Clinical Trial. J. Hepatol..

[70] Avila M.A., Berasain C., Torres L., Martín-Duce A., Corrales F.J., Yang H., Prieto J., Lu S.C., Caballería J., Rodés J. (2000). Reduced mRNA Abundance of the Main Enzymes Involved in Methionine Metabolism in Human Liver Cirrhosis and Hepatocellular Carcinoma. J. Hepatol..

[71] Villanueva J.A., Halsted C.H. (2004). Hepatic Transmethylation Reactions in Micropigs with Alcoholic Liver Disease. Hepatology.

[72] Finkelstein J.D., Cello J.P., Kyle W.E. (1974). Ethanol-Induced Changes in Methionine Metabolism in Rat Liver. Biochem. Biophys. Res. Commun..

[73] Halsted C.H., Villanueva J., Chandler C.J., Stabler S.P., Allen R.H., Muskhelishvili L., James S.J., Poirier L. (1996). Ethanol Feeding of Micropigs Alters Methionine Metabolism and Increases Hepatocellular Apoptosis and Proliferation. Hepatology.

[74] Rodríguez-Agudo R., González-Recio I., Serrano-Maciá M., Bravo M., Petrov P., Blaya D., Herranz J.M., Mercado-Gómez M., Rejano-Gordillo C.M., Lachiondo-Ortega S. (2024). Anti-miR-873-5p Improves Alcohol-Related Liver Disease by Enhancing Hepatic Deacetylation via SIRT1. JHEP Rep..

[75] Halsted C.H., Medici V. (2011). Vitamin-Dependent Methionine Metabolism and Alcoholic Liver Disease. Adv. Nutr..

[76] Lu S.C., Huang Z.Z., Yang J.M., Tsukamoto H. (1999). Effect of Ethanol and High-Fat Feeding on Hepatic Gamma-Glutamylcysteine Synthetase Subunit Expression in the Rat. Hepatology.

[77] Mato J.M., Lu S.C. (2007). Role of S-Adenosyl-L-Methionine in Liver Health and Injury. Hepatology.

[78] Finkelstein J.D. (1990). Methionine Metabolism in Mammals. J. Nutr. Biochem..

[79] Mudd S.H., Poole J.R. (1975). Labile Methyl Balances for Normal Humans on Various Dietary Regimens. Metabolism.

[80] Reytor E., Pérez-Miguelsanz J., Alvarez L., Pérez-Sala D., Pajares M.A. (2009). Conformational Signals in the C-Terminal Domain of Methionine Adenosyltransferase I/III Determine Its Nucleocytoplasmic Distribution. FASEB J..

[81] Murray B., Peng H., Barbier-Torres L., Robinson A.E., Li T.W.H., Fan W., Tomasi M.L., Gottlieb R.A., Van Eyk J., Lu Z. (2019). Methionine Adenosyltransferase A1 Is Targeted to the Mitochondrial Matrix and Interacts with Cytochrome P450 2E1 to Lower Its Expression. Hepatology.

[82] Watson W.H., Zhao Y., Chawla R.K. (1999). S-Adenosylmethionine Attenuates the Lipopolysaccharide-Induced Expression of the Gene for Tumour Necrosis Factor Alpha. Biochem. J..

[83] Ara A.I., Xia M., Ramani K., Mato J.M., Lu S.C. (2008). S-Adenosylmethionine Inhibits Lipopolysaccharide-Induced Gene Expression via Modulation of Histone Methylation. Hepatology.

[84] Yang H., Sadda M.R., Li M., Zeng Y., Chen L., Bae W., Ou X., Runnegar M.T., Mato J.M., Lu S.C. (2004). S-Adenosylmethionine and Its Metabolite Induce Apoptosis in HepG2 Cells: Role of Protein Phosphatase 1 and Bcl-x(S). Hepatology.

[85] Lu S.C., Alvarez L., Huang Z.Z., Chen L., An W., Corrales F.J., Avila M.A., Kanel G., Mato J.M. (2001). Methionine Adenosyltransferase 1A Knockout Mice Are Predisposed to Liver Injury and Exhibit Increased Expression of Genes Involved in Proliferation. Proc. Natl. Acad. Sci. USA.

[86] Martínez-Chantar M.L., Corrales F.J., Martínez-Cruz L.A., García-Trevijano E.R., Huang Z.-Z., Chen L., Kanel G., Avila M.A., Mato J.M., Lu S.C. (2002). Spontaneous Oxidative Stress and Liver Tumors in Mice Lacking Methionine Adenosyltransferase 1A. FASEB J..

[87] Santamaría E., Avila M.A., Latasa M.U., Rubio A., Martín-Duce A., Lu S.C., Mato J.M., Corrales F.J. (2003). Functional Proteomics of Nonalcoholic Steatohepatitis: Mitochondrial Proteins as Targets of S-Adenosylmethionine. Proc. Natl. Acad. Sci. USA.

[88] Robinson A.E., Binek A., Ramani K., Sundararaman N., Barbier-Torres L., Murray B., Venkatraman V., Kreimer S., Ardle A.M., Noureddin M. (2023). Hyperphosphorylation of Hepatic Proteome Characterizes Nonalcoholic Fatty Liver Disease in S-Adenosylmethionine Deficiency. iScience.

[89] Floris A., Chandla S., Lim Y., Barbier-Torres L., Seth K., Khangholi A., Li T.W.H., Robison A., Murray B.J., Lee S. (2024). Sumoylation of Methionine Adenosyltransferase Alpha 1 Promotes Mitochondrial Dysfunction in Alcohol-Associated Liver Disease. Hepatology.

[90] Tomasi M.L., Iglesias-Ara A., Yang H., Ramani K., Feo F., Pascale M.R., Martínez-Chantar M.L., Mato J.M., Lu S.C. (2009). S-Adenosylmethionine Regulates Apurinic/Apyrimidinic Endonuclease 1 Stability: Implication in Hepatocarcinogenesis. Gastroenterology.

[91] Rountree C.B., Senadheera S., Mato J.M., Crooks G.M., Lu S.C. (2008). Expansion of Liver Cancer Stem Cells during Aging in Methionine Adenosyltransferase 1A-Deficient Mice. Hepatology.

[92] Tomasi M.L., Ramani K., Lopitz-Otsoa F., Rodríguez M.S., Li T.W.H., Ko K., Yang H., Bardag-Gorce F., Iglesias-Ara A., Feo F. (2010). S-Adenosylmethionine Regulates Dual-Specificity Mitogen-Activated Protein Kinase Phosphatase Expression in Mouse and Human Hepatocytes. Hepatology.

[93] Vázquez M., Ariz U., Varela-Rey M., Embade N., Martínez N., Fernández D., Gómez L., Lamas S., Lu S.C., Martínez-Chantar M.L. (2009). Evidence for an LKB1/AMPK/eNOS Cascade Regulated by HGF, S-Adenosylmethionine and NO in Hepatocyte Proliferation. Hepatology.

[94] Alonso C., Fernández-Ramos D., Varela-Rey M., Martínez-Arranz I., Navasa N., Van Liempd S.M., Lavín Trueba J.L., Mayo R., Ilisso C.P., de Juan V.G. (2017). Metabolomic Identification of Subtypes of Nonalcoholic Steatohepatitis. Gastroenterology.

[95] Alarcón-Vila C., Insausti-Urkia N., Torres S., Segalés-Rovira P., Conde de la Rosa L., Nuñez S., Fucho R., Fernández-Checa J.C., García-Ruiz C. (2023). Dietary and Genetic Disruption of Hepatic Methionine Metabolism Induce Acid Sphingomyelinase to Promote Steatohepatitis. Redox Biol..

[96] Barić I., Erdol S., Saglam H., Lovrić M., Belužić R., Vugrek O., Blom H.J., Fumić K. (2016). Glycine N-Methyltransferase Deficiency: A Member of Dysmethylating Liver Disorders?. JIMD Rep..

[97] Andringa K.K., King A.L., Eccleston H.B., Mantena S.K., Landar A., Jhala N.C., Dickinson D.A., Squadrito G.L., Bailey S.M. (2010). Analysis of the Liver Mitochondrial Proteome in Response to Ethanol and S-Adenosylmethionine Treatments: Novel Molecular Targets of Disease and Hepatoprotection. Am. J. Physiol. Gastrointest. Liver Physiol..

[98] Chawla R.K., Hussain S., Watson W.H., Jones D.P. (1992). Effect of Ethanol Consumption on Metabolism of S-Adenosyl-L-Methionine in Rat Liver. Drug Investig..

[99] Sykora P., Kharbanda K.K., Crumm S.E., Cahill A. (2009). S-Adenosyl-L-Methionine Co-Administration Prevents the Ethanol-Elicited Dissociation of Hepatic Mitochondrial Ribosomes in Male Rats. Alcohol. Clin. Exp. Res..

[100] Aleynik S.I., Lieber C.S. (2003). Polyenylphosphatidylcholine Corrects The Alcohol-Induced Hepatic Oxidative Stress By Restoring S-Adenosylmethionine. Alcohol. Alcohol..

[101] Watson W.H., Song Z., Kirpich I.A., Deaciuc I.V., Chen T., McClain C.J. (2011). Ethanol Exposure Modulates Hepatic S-Adenosylmethionine and S-Adenosylhomocysteine Levels in the Isolated Perfused Rat Liver through Changes in the Redox State of the NADH/NAD+ System. Biochim. et Biophys. Acta (BBA) Mol. Basis Dis..

[102] Song Z., Zhou Z., Song M., Uriarte S., Chen T., Deaciuc I., McClain C.J. (2007). Alcohol-Induced S-Adenosylhomocysteine Accumulation in the Liver Sensitizes to TNF Hepatotoxicity: Possible Involvement of Mitochondrial S-Adenosylmethionine Transport. Biochem. Pharmacol..

[103] Prudova A., Bauman Z., Braun A., Vitvitsky V., Lu S.C., Banerjee R. (2006). S-Adenosylmethionine Stabilizes Cystathionine β-Synthase and Modulates Redox Capacity. Proc. Natl. Acad. Sci. USA.

[104] Barbier-Torres L., Murray B., Yang J.W., Wang J., Matsuda M., Robinson A., Binek A., Fan W., Fernández-Ramos D., Lopitz-Otsoa F. (2022). Depletion of Mitochondrial Methionine Adenosyltransferase A1 Triggers Mitochondrial Dysfunction in Alcohol-Associated Liver Disease. Nat. Commun..

[105] Schober F.A., Moore D., Atanassov I., Moedas M.F., Clemente P., Végvári Á., Fissi N.E., Filograna R., Bucher A.-L., Hinze Y. (2021). The One-Carbon Pool Controls Mitochondrial Energy Metabolism via Complex I and Iron-Sulfur Clusters. Sci. Adv..

[106] Vance D.E., Walkey C.J., Cui Z. (1997). Phosphatidylethanolamine N-Methyltransferase from Liver. Biochim. Biophys. Acta.

[107] Pérez C., Pérez-Zúñiga F.J., Garrido F., Reytor E., Portillo F., Pajares M.A. (2016). The Oncogene PDRG1 Is an Interaction Target of Methionine Adenosyltransferases. PLoS ONE.

[108] Fan W., Yang H., Liu T., Wang J., Li T.W.H., Mavila N., Tang Y., Yang J., Peng H., Tu J. (2017). Prohibitin 1 Suppresses Liver Cancers Tumorigenesis in Mice and Human Hepatocellular and Cholangiocarcinoma Cells. Hepatology.

[109] Zeng Z., Huang Z.Z., Chen C., Yang H., Mao Z., Lu S.C. (2000). Cloning and Functional Characterization of the 5′-Flanking Region of Human Methionine Adenosyltransferase 1A Gene. Biochem. J..

[110] Ikeda R., Nishida T., Watanabe F., Shimizu-Saito K., Asahina K., Horikawa S., Teraoka H. (2008). Involvement of CCAAT/Enhancer Binding Protein-Beta (C/EBPbeta) in Epigenetic Regulation of Mouse Methionine Adenosyltransferase 1A Gene Expression. Int. J. Biochem. Cell Biol..

[111] Torres L., Avila M.A., Carretero M.V., Latasa M.U., Caballería J., López-Rodas G., Boukaba A., Lu S.C., Franco L., Mato J.M. (2000). Liver-Specific Methionine Adenosyltransferase MAT1A Gene Expression Is Associated with a Specific Pattern of Promoter Methylation and Histone Acetylation: Implications for MAT1A Silencing during Transformation. FASEB J..

[112] Liu T., Yang H., Fan W., Tu J., Li T.W.H., Wang J., Shen H., Yang J., Xiong T., Steggerda J. (2018). Mechanisms of MAFG Dysregulation in Cholestatic Liver Injury and Development of Liver Cancer. Gastroenterology.

[113] Yang H., Liu T., Wang J., Li T.W.H., Fan W., Peng H., Krishnan A., Gores G.J., Mato J.M., Lu S.C. (2016). Deregulated Methionine Adenosyltransferase A1, c-Myc, and Maf Proteins Together Promote Cholangiocarcinoma Growth in Mice and Humans(‡). Hepatology.

[114] Vázquez-Chantada M., Fernández-Ramos D., Embade N., Martínez-Lopez N., Varela-Rey M., Woodhoo A., Luka Z., Wagner C., Anglim P.P., Finnell R.H. (2010). HuR/Methyl-HuR and AUF1 Regulate the MAT Expressed during Liver Proliferation, Differentiation, and Carcinogenesis. Gastroenterology.

[115] Koturbash I., Melnyk S., James S.J., Beland F.A., Pogribny I.P. (2013). Role of Epigenetic and miR-22 and miR-29b Alterations in the Downregulation of Mat1a and Mthfr Genes in Early Preneoplastic Livers in Rats Induced by 2-Acetylaminofluorene. Mol. Carcinog..

[116] Yang H., Cho M.E., Li T.W.H., Peng H., Ko K.S., Mato J.M., Lu S.C. (2013). MicroRNAs Regulate Methionine Adenosyltransferase 1A Expression in Hepatocellular Carcinoma. J. Clin. Investig..

[117] Stoyanov E., Mizrahi L., Olam D., Schnitzer-Perlman T., Galun E., Goldenberg D.S. (2017). Tumor-Suppressive Effect of S-Adenosylmethionine Supplementation in a Murine Model of Inflammation-Mediated Hepatocarcinogenesis Is Dependent on Treatment Longevity. Oncotarget.

[118] Sánchez-Góngora E., Ruiz F., Mingorance J., An W., Corrales F.J., Mato J.M. (1997). Interaction of Liver Methionine Adenosyltransferase with Hydroxyl Radical. FASEB J..

[119] Ruiz F., Corrales F.J., Miqueo C., Mato J.M. (1998). Nitric Oxide Inactivates Rat Hepatic Methionine Adenosyltransferase In Vivo by S-Nitrosylation. Hepatology.

[120] Avila M.A., Mingorance J., Martínez-Chantar M.L., Casado M., Martin-Sanz P., Boscá L., Mato J.M. (1997). Regulation of Rat Liver S-Adenosylmethionine Synthetase during Septic Shock: Role of Nitric Oxide. Hepatology.

[121] Pajares M.A., Durán C., Corrales F., Mato J.M. (1994). Protein Kinase C Phosphorylation of Rat Liver S-Adenosylmethionine Synthetase: Dissociation and Production of an Active Monomer. Biochem. J..

[122] Lu L., Zhang J., Fan W., Li Y., Wang J., Li T.W.H., Barbier-Torres L., Mato J.M., Liu T., Seki E. (2021). Deregulated 14-3-3ζ and Methionine Adenosyltransferase A1 Interplay Promotes Liver Cancer Tumorigenesis in Mice and Humans. Oncogene.

[123] Lieber C.S., Robins S.J., Leo M.A. (1994). Hepatic Phosphatidylethanolamine Methyltransferase Activity Is Decreased by Ethanol and Increased by Phosphatidylcholine. Alcohol. Clin. Exp. Res..

[124] Kharbanda K.K. (2009). Alcoholic Liver Disease and Methionine Metabolism. Semin. Liver Dis..

[125] Halsted C.H., Villanueva J.A., Devlin A.M., Niemelä O., Parkkila S., Garrow T.A., Wallock L.M., Shigenaga M.K., Melnyk S., James S.J. (2002). Folate Deficiency Disturbs Hepatic Methionine Metabolism and Promotes Liver Injury in the Ethanol-Fed Micropig. Proc. Natl. Acad. Sci. USA.

[126] Lambert M.-P., Paliwal A., Vaissière T., Chemin I., Zoulim F., Tommasino M., Hainaut P., Sylla B., Scoazec J.-Y., Tost J. (2011). Aberrant DNA Methylation Distinguishes Hepatocellular Carcinoma Associated with HBV and HCV Infection and Alcohol Intake. J. Hepatol..

[127] Medici V., Schroeder D.I., Woods R., LaSalle J.M., Geng Y., Shibata N.M., Peerson J., Hodzic E., Dayal S., Tsukamoto H. (2014). Methylation and Gene Expression Responses to Ethanol Feeding and Betaine Supplementation in the Cystathionine Beta Synthase-Deficient Mouse. Alcohol. Clin. Exp. Res..

[128] Hlady R.A., Tiedemann R.L., Puszyk W., Zendejas I., Roberts L.R., Choi J.-H., Liu C., Robertson K.D. (2014). Epigenetic Signatures of Alcohol Abuse and Hepatitis Infection during Human Hepatocarcinogenesis. Oncotarget.

[129] Yuan G.-J., Zhou X.-R., Gong Z.-J., Zhang P., Sun X.-M., Zheng S.-H. (2006). Expression and Activity of Inducible Nitric Oxide Synthase and Endothelial Nitric Oxide Synthase Correlate with Ethanol-Induced Liver Injury. World J. Gastroenterol..

[130] Shankarappa B., Mahadevan J., Murthy P., Purushottam M., Viswanath B., Jain S., Devarbhavi H., Mysore Visweswariah A. (2023). Hypomethylation of Long Interspersed Nucleotide Elements and Aldehyde Dehydrogenase in Patients of Alcohol Use Disorder with Cirrhosis. DNA Cell Biol..

[131] Wang Y., Zhang S., Xie X., Chen Z., Wu L., Yu Z., Guo X., Chen G. (2020). Association of TNFRSF12A Methylation With Prognosis in Hepatocellular Carcinoma With History of Alcohol Consumption. Front. Genet..

[132] Esfandiari F., Villanueva J.A., Wong D.H., French S.W., Halsted C.H. (2005). Chronic Ethanol Feeding and Folate Deficiency Activate Hepatic Endoplasmic Reticulum Stress Pathway in Micropigs. Am. J. Physiol. Gastrointest. Liver Physiol..

[133] Esfandiari F., You M., Villanueva J.A., Wong D.H., French S.W., Halsted C.H. (2007). S-Adenosylmethionine Attenuates Hepatic Lipid Synthesis in Micropigs Fed Ethanol with a Folate-Deficient Diet. Alcohol. Clin. Exp. Res..

[134] Kusumanchi P., Liang T., Zhang T., Ross R.A., Han S., Chandler K., Oshodi A., Jiang Y., Dent A.L., Skill N.J. (2021). Stress-Responsive Gene FK506-Binding Protein 51 Mediates Alcohol-Induced Liver Injury Through the Hippo Pathway and Chemokine (C-X-C Motif) Ligand 1 Signaling. Hepatology.

[135] Zeybel M., Hardy T., Robinson S.M., Fox C., Anstee Q.M., Ness T., Masson S., Mathers J.C., French J., White S. (2015). Differential DNA Methylation of Genes Involved in Fibrosis Progression in Non-Alcoholic Fatty Liver Disease and Alcoholic Liver Disease. Clin. Epigenetics.

[136] Shen H., French B.A., Tillman B.C., Li J., French S.W. (2015). Increased DNA Methylation in the Livers of Patients with Alcoholic Hepatitis. Exp. Mol. Pathol..

[137] Powell C.L., Bradford B.U., Craig C.P., Tsuchiya M., Uehara T., O’Connell T.M., Pogribny I.P., Melnyk S., Koop D.R., Bleyle L. (2010). Mechanism for Prevention of Alcohol-Induced Liver Injury by Dietary Methyl Donors. Toxicol. Sci..

[138] Cruise T.M., Kotlo K., Malovic E., Pandey S.C. (2023). Advances in DNA, Histone, and RNA Methylation Mechanisms in the Pathophysiology of Alcohol Use Disorder. Adv. Drug Alcohol. Res..

[139] Pal-Bhadra M., Bhadra U., Jackson D.E., Mamatha L., Park P.-H., Shukla S.D. (2007). Distinct Methylation Patterns in Histone H3 at Lys-4 and Lys-9 Correlate with up- & down-Regulation of Genes by Ethanol in Hepatocytes. Life Sci..

[140] Shukla S.D., Lim R.W. (2013). Epigenetic Effects of Ethanol on the Liver and Gastrointestinal System. Alcohol. Res..

[141] Bardag-Gorce F., Oliva J., Dedes J., Li J., French B.A., French S.W. (2009). Chronic Ethanol Feeding Alters Hepatocyte Memory Which Is Not Altered by Acute Feeding. Alcohol. Clin. Exp. Res..

[142] Veal N., Hsieh C.-L., Xiong S., Mato J.M., Lu S., Tsukamoto H. (2004). Inhibition of Lipopolysaccharide-Stimulated TNF-Alpha Promoter Activity by S-Adenosylmethionine and 5′-Methylthioadenosine. Am. J. Physiol. Gastrointest. Liver Physiol..

[143] Meng F., Glaser S.S., Francis H., Yang F., Han Y., Stokes A., Staloch D., McCarra J., Liu J., Venter J. (2012). Epigenetic Regulation of miR-34a Expression in Alcoholic Liver Injury. Am. J. Pathol..

[144] Barcena-Varela M., Colyn L., Fernandez-Barrena M.G. (2019). Epigenetic Mechanisms in Hepatic Stellate Cell Activation During Liver Fibrosis and Carcinogenesis. Int. J. Mol. Sci..

[145] Matsui H., Kawada N. (2005). Effect of S-Adenosyl-L-Methionine on the Activation, Proliferation and Contraction of Hepatic Stellate Cells. Eur. J. Pharmacol..

[146] Zhang F., Zhuge Y.-Z., Li Y.-J., Gu J.-X. (2014). S-Adenosylmethionine Inhibits the Activated Phenotype of Human Hepatic Stellate Cells via Rac1 and Matrix Metalloproteinases. Int. Immunopharmacol..

[147] Karaa A., Thompson K.J., McKillop I.H., Clemens M.G., Schrum L.W. (2008). S-Adenosyl-L-Methionine Attenuates Oxidative Stress and Hepatic Stellate Cell Activation in an Ethanol-LPS-Induced Fibrotic Rat Model. Shock.

[148] Thompson K.J., Lakner A.M., Cross B.W., Tsukada S., Rippe R.A., McKillop I.H., Schrum L.W. (2011). S-Adenosyl-L-Methionine Inhibits Collagen Secretion in Hepatic Stellate Cells via Increased Ubiquitination. Liver Int..

[149] Walker A.K. (2017). 1-Carbon Cycle Metabolites Methylate Their Way to Fatty Liver. Trends Endocrinol. Metab..

[150] Barbier-Torres L., Chhimwal J., Kim S.Y., Ramani K., Robinson A., Yang H., Van Eyk J., Liangpunsakul S., Seki E., Mato J.M. (2024). S-Adenosylmethionine Negatively Regulates the Mitochondrial Respiratory Chain Repressor MCJ in the Liver. Int. J. Biol. Sci..

[151] Glasgow R.I.C., Singh V., Peña-Pérez L., Wilhalm A., Moedas M.F., Moore D., Rosenberger F.A., Li X., Atanassov I., Saba M. (2025). The Mitochondrial Methylation Potential Gates Mitoribosome Assembly. Nat. Commun..

[152] Zhao X.-J., Dong Q., Bindas J., Piganelli J.D., Magill A., Reiser J., Kolls J.K. (2008). TRIF and IRF-3 Binding to the TNF Promoter Results in Macrophage TNF Dysregulation and Steatosis Induced by Chronic Ethanol. J. Immunol..

[153] Thakur V., Pritchard M.T., McMullen M.R., Wang Q., Nagy L.E. (2006). Chronic Ethanol Feeding Increases Activation of NADPH Oxidase by Lipopolysaccharide in Rat Kupffer Cells: Role of Increased Reactive Oxygen in LPS-Stimulated ERK1/2 Activation and TNF-α Production. J. Leukoc. Biol..

[154] Chawla R.K., Watson W.H., Eastin C.E., Lee E.Y., Schmidt J., McClain C.J. (1998). S-Adenosylmethionine Deficiency and TNF-Alpha in Lipopolysaccharide-Induced Hepatic Injury. Am. J. Physiol..

[155] Mansouri A., Gaou I., De Kerguenec C., Amsellem S., Haouzi D., Berson A., Moreau A., Feldmann G., Lettéron P., Pessayre D. (1999). An Alcoholic Binge Causes Massive Degradation of Hepatic Mitochondrial DNA in Mice. Gastroenterology.

[156] Chedid A., Mendenhall C.L., Tosch T., Chen T., Rabin L., Garcia-Pont P., Goldberg S.J., Kiernan T., Seeff L.B., Sorrell M. (1986). Significance of Megamitochondria in Alcoholic Liver Disease. Gastroenterology.

[157] Hao L., Zhong W., Dong H., Guo W., Sun X., Zhang W., Yue R., Li T., Griffiths A., Ahmadi A.R. (2021). ATF4 Activation Promotes Hepatic Mitochondrial Dysfunction by Repressing NRF1-TFAM Signaling in Alcoholic Steatohepatitis. Gut.

[158] King A.L., Swain T.M., Dickinson D.A., Lesort M.J., Bailey S.M. (2010). Chronic Ethanol Consumption Enhances Sensitivity to Ca(2+)-Mediated Opening of the Mitochondrial Permeability Transition Pore and Increases Cyclophilin D in Liver. Am. J. Physiol. Gastrointest. Liver Physiol..

[159] Setshedi M., Wands J.R., de la Monte S.M. (2010). Acetaldehyde Adducts in Alcoholic Liver Disease. Oxid. Med. Cell Longev..

[160] Lluis J.M., Colell A., García–Ruiz C., Kaplowitz N., Fernández–Checa J.C. (2003). Acetaldehyde Impairs Mitochondrial Glutathione Transport in HepG2 Cells through Endoplasmic Reticulum Stress. Gastroenterology.

[161] Cortés-Rojo C., Vargas-Vargas M.A., Olmos-Orizaba B.E., Rodríguez-Orozco A.R., Calderón-Cortés E. (2020). Interplay between NADH Oxidation by Complex I, Glutathione Redox State and Sirtuin-3, and Its Role in the Development of Insulin Resistance. Biochim. Biophys. Acta Mol. Basis Dis..

[162] You M., Matsumoto M., Pacold C.M., Cho W.K., Crabb D.W. (2004). The Role of AMP-Activated Protein Kinase in the Action of Ethanol in the Liver. Gastroenterology.

[163] Wang S., Wan T., Ye M., Qiu Y., Pei L., Jiang R., Pang N., Huang Y., Liang B., Ling W. (2018). Nicotinamide Riboside Attenuates Alcohol Induced Liver Injuries via Activation of SirT1/PGC-1α/Mitochondrial Biosynthesis Pathway. Redox Biol..

[164] Picklo M.J. (2008). Ethanol Intoxication Increases Hepatic N-Lysyl Protein Acetylation. Biochem. Biophys. Res. Commun..

[165] Decker S.T., Funai K. (2024). Mitochondrial Membrane Lipids in the Regulation of Bioenergetic Flux. Cell Metab..

[166] Park W.-J., Song J.-H., Kim G.-T., Park T.-S. (2020). Ceramide and Sphingosine 1-Phosphate in Liver Diseases. Mol. Cells.

[167] Watson W.H., Burke T.J., Doll M.A., McClain C.J. (2014). S-Adenosylhomocysteine Inhibits NFκB-Mediated Gene Expression in Hepatocytes and Confers Sensitivity to TNF Cytotoxicity. Alcohol. Clin. Exp. Res..

[168] Wang X., Cederbaum A.I. (2008). S-Adenosyl-L-Methionine Decreases the Elevated Hepatotoxicity Induced by Fas Agonistic Antibody plus Acute Ethanol Pretreatment in Mice. Arch. Biochem. Biophys..

[169] Gigliozzi A., Romeo R., Fraioli F., Cantafora A., Delle Monache M., Cardilli A., Attili A.F., Scafato E., Carli L., Alvaro D. (1998). Effect of S-Adenosyl-L-Methionine and Dilinoleoylphosphatidylcholine on Liver Lipid Composition and Ethanol Hepatotoxicity in Isolated Perfused Rat Liver. Dig. Dis. Sci..

[170] Oliva J., Bardag-Gorce F., Li J., French B.A., French S.W. (2011). S-Adenosylmethionine Prevents the up Regulation of Toll-like Receptor (TLR) Signaling Caused by Chronic Ethanol Feeding in Rats. Exp. Mol. Pathol..

[171] Ji C., Kaplowitz N. (2003). Betaine Decreases Hyperhomocysteinemia, Endoplasmic Reticulum Stress, and Liver Injury in Alcohol-Fed Mice. Gastroenterology.

[172] Kharbanda K.K., Todero S.L., King A.L., Osna N.A., McVicker B.L., Tuma D.J., Wisecarver J.L., Bailey S.M. (2012). Betaine Treatment Attenuates Chronic Ethanol-Induced Hepatic Steatosis and Alterations to the Mitochondrial Respiratory Chain Proteome. Int. J. Hepatol..

[173] Bingül İ., Başaran-Küçükgergin C., Aydın A.F., Çoban J., Doğan-Ekici I., Doğru-Abbasoğlu S., Uysal M. (2016). Betaine Treatment Decreased Oxidative Stress, Inflammation, and Stellate Cell Activation in Rats with Alcoholic Liver Fibrosis. Environ. Toxicol. Pharmacol..

[174] Arumugam M.K., Chava S., Perumal S.K., Paal M.C., Rasineni K., Ganesan M., Donohue T.M., Osna N.A., Kharbanda K.K. (2022). Acute Ethanol-Induced Liver Injury Is Prevented by Betaine Administration. Front. Physiol..

[175] Vendemiale G., Altomare E., Trizio T., Le Grazie C., Di Padova C., Salerno M.T., Carrieri V., Albano O. (1989). Effects of Oral S-Adenosyl-L-Methionine on Hepatic Glutathione in Patients with Liver Disease. Scand. J. Gastroenterol..

[176] Medici V., Virata M.C., Peerson J.M., Stabler S.P., French S.W., Gregory J.F., Albanese A., Bowlus C.L., Devaraj S., Panacek E.A. (2011). S-Adenosyl-L-Methionine Treatment for Alcoholic Liver Disease: A Double-Blinded, Randomized, Placebo-Controlled Trial. Alcohol. Clin. Exp. Res..

